# Measuring Support for Women’s Political Leadership: Gender of Interviewer Effects Among African Survey Respondents

**DOI:** 10.1093/poq/nfac031

**Published:** 2022-09-02

**Authors:** Aksel Sundström, Daniel Stockemer

**Affiliations:** Aksel Sundström is an associate professor in the Department of Political Science at the University of Gothenburg, Sweden; Daniel Stockemer is Konrad Adenauer Research Chair in Empirical Democracy Studies and professor in the School of Political Studies at the University of Ottawa, Canada

## Abstract

Public opinion surveys are a fundamental tool to measure support for women’s political rights. This article focuses on perceptions of women’s suitability for leadership. To what extent do influential cross-country surveys that include such items suffer from measurement errors stemming from gender of interviewer effects? Building on the literature on social desirability, we expect that respondents are more likely to express preference for men’s suitability as political leaders with male interviewers and more likely to state support for women’s leadership when interviewed by a woman. We hypothesize that these processes are conditioned by having one’s spouse present, by age differences between respondents and interviewers, as well as by respondents’ levels of education. Analyzing Afrobarometer data, we generally find support for our claims. In addition, it seems that men are slightly more affected by such effects than women are. These gender of interviewer effects persist when analyzing alternative survey rounds and are insensitive to various fixed effects specifications and robustness tests. For the analysis of survey data, we suggest that researchers using gender-related items should control for gender of interviewer effects. We propose that comparative survey programs pay even more attention to interviewer characteristics and the interview situation in their protocols.

## Introduction

Survey items gauging the extent to which citizens see women as fit for political leadership (Inglehart and Norris 2003) commonly capture public demand for women in public office (Lovenduski and Norris 1993). Opinions about women’s fitness for higher political office serve as proxy variables to understand gender equality in low-income countries (Bolzendahl and Myers 2004), especially where women’s rights are weak (Benstead 2018a, 2018b). This article looks at gendered distortions in citizens’ assessment of women’s suitability to hold higher political office, focusing on the sex of the interviewer. To what extent do men and women give different answers to questions about women’s fitness for political office when interviewed by either a male interviewer or a female interviewer, and to what extent do influential cross-country surveys suffer from such measurement errors?

Initially based on small samples within the United States (Benney et al. 1956; Landis et al. 1973) and more recently on representative samples in single-country settings (e.g., Flores-Macias and Lawson 2008; Benstead 2014a), empirical work on gender of interviewer effects has not yet explored interviewer effects on gender-related items in cross-county surveys. Therefore, the literature has seldom discussed the “big picture,” that is, how such potential measurement errors affect our understanding of support for women’s rights across the globe or in one region. For instance, the largest comparative survey program, the World Values Survey, has never systematically recorded the gender of interviewers.

Social desirability—where respondents seek to make a favorable impression and adopt their answers to the interviewer’s expected opinions—could explain why survey participants give a different answer when interviewed by a man rather than a woman. In such interactions, the respondent might align their views to the perceived views of the interviewer and adjust their responses in the direction of what they think the interviewer wants to hear. When the interviewer and the respondent are the same genders, self-disclosure theories imply that respondents are less likely to shy away from revealing sensitive attitudes (see Catania et al. 1996; Dykema et al. 2012), allowing men possibilities to air attitudes about men’s superiority and giving women room to voice attitudes about equal rights. Yet, these same-sex dyads are possibly also tainted with socially desirable responding, as respondents could act to reinforce in-group esteem by answering in line with the stereotyped views of their gender (Benstead 2014b). This leads us to expect that male and female respondents are more prone to express preference for male leadership with male interviewers and less prone when interviewed by a woman. Moreover, we believe that the presence of one’s spouse, the age difference between respondent and interviewer, and the education level of the respondent could condition these interviewer effects. We test our expectations in an analysis of round six of the Afrobarometer project.

## Interviewer Effects

The study of public opinion in low-income countries is beset with several methodological challenges (Lupu and Michelicht 2018). Much of today’s survey research in such settings builds on face-to-face interviews, situations where interviewer effects could matter. Aware of these potential effects, prior research has looked at how a range of observable interviewer characteristics, such as linkages between the audio-visual (e.g., skin color, a gendered voice, and accents) and social categorizations (e.g., social class and race), systematically alter answers from respondents (Hyman et al. 1954; Schuman and Converse 1971; Campbell 1981).^1^ In particular, there is some consensus that appearance-based characteristics such as the wearing of a headscarf can affect answers in the field of gender equality (Blaydes and Gillum 2013; Benstead 2014a, 2014b). Another relevant finding, from the context of African countries, is that co-ethnicity matters: respondents are more likely to voice discriminatory attitudes toward other ethnic groups when interviewed by someone of their own ethnicity (Adida et al. 2016).

## Gender of Interviewer Effects

The gender of the interviewer can also play an important role in the interview process (Johnson and Braun 2016). In fact, studies on gender of interviewer effects build on three bodies of literature: research on 1) survey interviews, 2) job interviews and counselling studies, and 3) social-psychological experiments (Lueptow et al. 1990). For example, one vein of work proposes that female interviewers obtain better response rates (Benney et al. 1956) and quality of responses (Liu and Wang 2016), because they appear less threatening and are more likely to gain access to respondents’ homes (Huddy et al. 1997), even if this effect is not always significant (West and Blom 2017). Another feature, discussed by Becker, Feyisetan, and Makinwa-Adebusoye (1995), refers to the tendency of female respondents to shy away from answering questions about sexual behavior when interviewed by a man (see also Pollner 1998; Davis et al. 2010).

Does the interviewer’s gender influence attitudes toward gender equality among male and female respondents? Landis et al. (1973) analyzed a sample of US students’ responses to issues related to gender roles in society and found that female students give more feminist responses when interviewed by women. Studying a small group of US students, Lueptow and colleagues (1990) add that women voice more liberal attitudes to female interviewers. Another study, by Kane and Macaulay (1993), establishes that men state different attitudes about gender inequalities in the labor market, when interviewed by men and women, respectively (see also Catania et al. 1996). Huddy and colleagues (1997) add that these trends are larger among the less well-educated and younger respondents.

Social-psychological experiments further suggest that gender might be a cue for what the respondents perceive as a desirable response. For instance, Galla et al. (1981) find that male interviewers generate more traditional attitudes from female respondents on a sex-role questionnaire (see also Frisone et al. 1982). Work by Flores-Macias and Lawson (2008) also reports gender of interviewer effects for questions on women’s rights among men, but only in the part of their sample that is drawn from the capital region in Mexico, where respondents’ characteristics might be more heterogeneous than in more rural parts of the country. Finally, in the Moroccan context, Benstead (2014a) finds that interviewers’ visible religiosity and gender interactively affect responses to religiously sensitive questions.

## Theoretical Expectations

We know from a case study of Morocco (see Benstead 2014b) that men tend to report more egalitarian answers to questions about women and politics when interviewed by women. Yet, it is not clear from this case study whether these effects are generalizable to other contexts and, if any, which other factors condition these processes. To understand these effects theoretically, we make use of the literature on social desirability, which Fisher (1993, p. 303) defines as “systematic error in self-report measures resulting from the desire of respondents to avoid embarrassment and project a favorable image to others.” In detail, processes of social desirability generally reflect people’s propensity to “deny socially undesirable traits and to claim socially desirable ones, and the tendency to say things which place the speaker in a favorable light” (Nederhof 1985, p. 264). Face-to-face interviews are particularly prone to social desirability biases. To explain the origin of these predispositions, Brenner (2017, p. 7) suggests that observable characteristics are important in interactions where you have no experience of working together: “the dyad—interviewer and sample element—begins an interaction typically with very little information about the other. [They] quickly size up each other on the basis of physical appearance, vocal characteristics, and so on.” Such interactions trigger beliefs about what is desirable and are heterogonous across survey item types: “Respondents tend to report attitudes in line with their expectation of the interviewer’s opinion on the basis of these observable characteristics … [but] only those questions relevant to the observable physical characteristic are prone to interviewer effects” (Brenner 2017, p. 6). In addition, social desirability is likely to appear in discussions about controversial political questions, when respondents might “believe their true answer goes against perceived societal norms” (Streb et al. 2008, p. 77).

The notion of social distance—a concept that pinpoints how similarities in characteristics between interviewer and interviewee (such as gender or race) determines perceptions of whether the two actors share a mutual understanding of different phenomena (Tu and Liao 2007)—can explain why respondents engage in socially desirable responding. Shying away from statements that could contradict the interviewer’s believed opinions can be a mechanism to reduce the social distance between interviewer and respondent (Williams 1964; Landis et al. 1973; Benstead 2014a). These processes follow what Paulhus (2002) labels impression management and self-deception. As Nederhof (1985, p. 264) notes, impression management is about the “norms of what constitutes a good impression in a given situation.”^2^

It is plausible that processes related to impression management apply to both male and female respondents, especially in the African context, where women’s political rights are far from taken for granted (see Sundström et al. 2017) and where voicing ideas about gender equality might still be radical. Deference theory or power relations theory, which builds on the reasoning that constructed gender roles tend to shape how people behave in conversations (Lau 2018), would predict that women could have reasons to downplay gender equality views when interviewed by a man. Being outspoken about the promotion of women’s rights might require considerable self-confidence. In line with this view, theories on attribution further suggest that women might attribute less progressive gender-related views to men when trying to adjust in a socially desirable way to their male conversation partners (Benney et al. 1956; Hyman 1954). This implies that respondents who feel subordinate to the interviewer may be more likely to engage in socially desirable responding and adjust their stated views to the perceived opinions of the interviewer. In such a situation, a woman describing herself as a feminist might still expect to receive ridicule or hostile responses from men. As a result, we expect that women tend to voice more gender-egalitarian opinions to a woman than to a man.

Similar interviewer effects should be at play for men. For example, theories of self-disclosure could explain men’s tendencies to prefer male leaders when interviewed by men. According to this reasoning, survey respondents are more likely to expose sensitive views to an interviewer perceived as supportive or non-judgmental (Catania et al. 1996; Lau 2018). In such a situation, the processes of impression management and self-deception would not kick into place. As stated by Dykema and colleagues (2012, p. 312), “individuals are expected to be more honest and disclose more to someone they trust and with whom they feel comfortable.”^3^ However, this is not to say that the responses in same-sex interviews are completely free from processes where respondents adjust according to who is asking the questions. In fact, Benstead (2014a) notes that “respondents (might) demonstrate loyalty and enhance in-group esteem by agreeing with the stereotyped views of their in-group” (pp. 740–44). They might do so to impress upon their conversation partner or because they feel comfortable to voice such opinions. Regardless, attitudes of preferences for male leadership should be more likely to emerge in conversations among only male participants. Male pairs may invoke social pressures for respondents to engage in “locker room talk” in which they can air attitudes about female subordination freely and even in exaggerated ways (Grenz 2005). In contrast, men might voice more egalitarian views when it comes to women leadership roles when the interviewer is a woman. In the words of Streb et al. (2008, p. 79), “respondents might want to avoid appearing sexist” when talking to a woman. Taken together, this leads to the following expectation:

*Hypothesis 1:* Respondents are more likely to state support for the election of women to political leadership positions when interviewed by a woman than when interviewed by a man. Likewise, they are less likely to state that men are better suited for leadership when interviewed by a woman than when interviewed by a man.

While this hypothesis predicts that both men and women are susceptible to interviewer effects, it is also important to detect if the size of these effects differs between the two groups (see Huddy et al. 1997). Methodologically, more susceptibility to interviewer effects by either men or women would entail more noise in the answers of this particular group. Greater gender of interviewer effects for women would be consistent with the theoretical idea of “sex role stereotyping, where females are generally more sensitive to the characteristics of the interview situation, especially when these involve threat or desirability” (Lueptow et al. 1990, p. 31; see also Kane and Macaulay 1993). In contrast, greater gender of interviewer effects for men would imply that men, on average, still adhere to gender-traditional attitudes, but are more likely to voice these thoughts when being interviewed by a man (Flores-Macias and Lawson 2008). Since we do not know a priori which of the two expectations is more in tune with reality, we postulate the two following hypotheses:

*Hypothesis 2a*: Gender of interviewer effects on survey items gauging stated support for women in political leadership are larger among women than among men.

*Hypothesis 2b*: Gender of interviewer effects on survey items gauging stated support for women in political leadership are larger among men than among women.

Gender of interviewer effects might also depend on the privacy of the interview or whether other parties, especially spouses, are present (Zipp and Toth 2002). Hartmann (1994) suggests that privacy matters in the interview, which often takes place in the household. For example, the presence of a “third party,” which may include spouses, children, or bystanders, can influence respondents’ answers on sensitive issues such as gender roles (Smith 1997; Mneimneh et al. 2018). We deem it likely that the presence of the interviewee’s husband or wife could subtly impede the airing of traditional attitudes (among men) or possibly defiant views (among women) about the role of women in political office.^4^

There are four possible dyads or combinations between the gender of the interviewer and the respondent. In a male-male dyad, the presence of the respondent’s wife could instill some restraint, making men uncomfortable to belittle women’s rights. With a female interviewer, a male respondent with his wife present could be even more reluctant to express aversion toward having female leaders; the presence of two women might be a double constraint for men to voice preferences for male leadership. In the third combination, a female-female dyad, the presence of the husband might dampen the female respondent’s tendency to express emancipatory views. She might feel uneasy to state egalitarian views because her husband could disapprove. With a male interviewer, the effect of a husband’s presence is plausibly stronger; in the presence of two men, a female respondent might not dare to express progressive views about women’s political rights.

*Hypothesis 3*: The presence of the spouse will weaken gender of interviewer effects on survey items gauging stated support for women in political leadership, and this weakening effect will be particularly strong in mixed-sex interviewer/respondent dyads.

In the context of an interview, it is possible that the age difference between interviewer and interviewee can influence respondents’ view of socially acceptable opinions (Lau 2018). We believe that stating progressive opinions to an older interviewer can feel intimidating, because somebody might worry about how it makes her appear to that older person, especially in a context where respect for older people is more ingrained than in European or North American settings. This might particularly apply to a woman if the conversation partner is an older man. More generally, because of deference, the presence of an older interviewer could increase men’s tendency to state support for traditional gender roles and therefore strengthen the magnitude of gender of interviewer effects on gendered issues. Even more so, to be interviewed by a much older person may decrease women’s likelihood to voice gender equality attitudes, and this should interact with the gender of the interviewer.

*Hypothesis 4*: Gender of interviewer effects on survey items gauging stated support for women in political leadership will intensify when the interviewer is considerably older than the respondent, particularly if the interviewer is a man.

There is an extensive literature (Schuman and Converse 1971; Campbell 1981; Huddy et al. 1997; Blaydes and Gillum 2013) that finds that less well-educated individuals seem to be more easily swayed by social desirability and interviewer effects in surveys. Applied to support for women’s political rights, this would imply that individuals with less education should have a higher tendency to “please” the interviewer by voicing preferences for male leadership if the interviewer is a man and a more emancipatory stance if the interviewer is a woman.

*Hypothesis 5*: Gender of interviewer effects on survey items gauging stated support for women in political leadership will be smaller at higher levels of respondent education.

## Research Design

We focus on round six of the Afrobarometer project, which was collected in 2014 to 2015 and covers 36 countries.^5^ The Afrobarometer survey draws a clustered, stratified, multi-stage area probability sample that consists of 49.8 percent men (and 50.2 percent women).^6^ In-person interviews are conducted in respondents’ households. We are confident that gender of interviewer effects in the data do not stem from assignment bias or a systematic skewness in the assignment of male or female interviewers to areas and respondents (Flores-Macias and Lawson 2008; Lau 2018; Adida et al. 2016; Lupu and Michelicht 2018). The Afrobarometer protocol oversees the completion of the survey in the same way in all partner countries and the organization trains the national team, employed by a partner firm, in its implementation. When fielded, interviewers move in gender-mixed teams consisting of one field supervisor and four interviewers (Afrobarometer 2014, p. 6). Therefore, a team’s withdrawal from an insecure enumeration area will not disproportionally affect the areas surveyed by men or women. To corroborate this, we interviewed the Afrobarometer Project’s Deputy Director of Surveys. We also communicated with 15 of the national partner firms, which all confirmed that teams indeed consist of both men and women (see Supplemental Material table Sm2 for details and Supplemental Material table Sm3, which shows the gender distribution of interviewers per country).

Moreover, the assignment of interviewers to respondents should not create gendered distortions. The survey adheres to a gender quota in sampling: on any given day, an interviewer alternates respondents by gender, starting each morning by referring to her last interview the previous day, or a coin-flip.^7^ Upon approaching a selected household—and knowing which gender to sample—a list of potential respondents is established as the adult household member first encountered describes who in the household are women or men. The interviewer clarifies: “We would like to choose an adult from your household. Would you help us pick one?” One person from the allotted gender category is chosen by drawing a numbered card.^8^

## Dependent Variable

Our dependent variable measures attitudes about whether men make better political leaders than women, which we see as a fundamental hurdle for women’s advancement in the political sphere (Lovenduski and Norris 1993; Inglehart and Norris 2003). In detail, the interviewer reads aloud:

Which of the following statements is closest to your view? Choose Statement 1 or Statement 2.

Statement one: Men make better political leaders than women, and should be elected rather than women.

Statement two: Women should have the same chance of being elected to political office as men.

Interviewers record the neutral response “agree with neither,” even if they did not read it aloud. For respondents who selected one of the two statements, interviewers probe the strength of the opinion by asking, “Do you agree or agree very strongly?” Therefore, responses range from 0 (agree very strongly with statement one) to 4 (agree very strongly with statement two), with a neutral mid-category.^9^

## Gender of Respondent and Interviewer

To construct our key independent variable, we created four dummy variables, one for each of the following dyads: 1) the respondent and interviewer are both male, 2) the respondent is male and the interviewer is female, 3) the respondent is female and the interviewer is male, and 4) the respondent and interviewer are both female.

## Additional Independent Variables

Our full models have several independent variables. The indicators capturing education, also self-reported, consist of four dummy variables: *no formal education*, *primary education*, *secondary education*, and *post-secondary education*. Three dummy variables gauge privacy during the interview: a) if *no one else* were in the same room, b) if a *spouse only* was present, and c) if *others* were attending the interview (combining the options “children,” “a small crowd,” or “a few others”).^10^ Finally, we use information on age of respondents (self-reported, in years) and age of the interviewer (in years, recorded by the interviewer completing the survey), to gauge the *age difference* between interviewers and respondents. In more detail, we create a binary variable coded 1 if the interviewer is at least 15 years older than the interviewee (0 if not).^11^ We opt for the 15-year difference for two reasons. First, and more theoretically, 15 years is generally the lower limit to denote a generation (see Pew Research Center 2015). At the time of the survey (i.e., in 2014/2015), most respondents belonged to Generation X (those born between 1965 and 1980) and the millennial generation (those born between 1981 and 1997). To qualify for participation in the survey, participants had to be 18 years of age. This implies that the life span of these two most important generations in the survey was 15 years for Generation X, and 16 years for the millennials. More empirically, if we were to use a higher limit to denote a generation, we would have very few interviewer-respondent dyads with a sufficiently large age difference. To illustrate, there are only 2 percent of the dyads where the age difference is 20 years or more. We control for respondents’ residency and religious denomination to hold constant two factors that could determine variation in gender equality attitudes (Bolzendahl and Myers 2004). To capture residency, we add a dummy variable coded 1 for *urban* and 0 for *rural* (categories recorded by survey administrators). Religion distinguishes between *Christians*, *Muslims*, and *other religions* (stated information by respondents), through dummy variables. Table 1 reports summary statistics of all variables (for the exact question wording of the items we used, see Supplementary Material table Sm22).

**Table 1.** Summary statistics for main variables

Variables	%
Support for women leaders (N = 53,375)	
Very strongly agree with statement 1 (men better)	19.4
Agree with statement 1 (men better)	12.2
Agree with neither (volunteered)	1.5
Agree with statement 2 (women equal)	24.1
Very strongly agree with statement 2 (women equal)	43
Gender of respondent and interviewer (N = 53,935)	
Respondent male/interviewer male	25.4
Respondent male/interviewer female	25.1
Respondent female/interviewer male	24.3
Respondent female/interviewer female	25.2
Presence of others in the household (N = 53,808)	
No one present	65.9
Spouse present	7.4
Others present	26.6
Age difference of more than 15 years (N = 53,935)	4.4
Educational attainment (N = 53,780)	
No formal education	19
Primary education	29
Secondary education	36.2
Post-secondary education	15.8
Religion (N = 52,618)	
Christian	60.6
Muslim	29.7
Other religion	9.7
Locale (N = 53,315)	
Urban	57.9
Rural	42.1

Our analysis has several steps. First, to examine whether there are interviewer effects, we report cross-tabular statistics on the gendered leadership questions across the four dyads, and use a Chi-square test of independence to test whether differences are significant.^12^ Second, to test hypotheses 1 and 2, we evaluate gender of interviewer effects using multivariate regression.^13^ On the left-hand side of Model 1 is the five-value ordinal gendered leadership variable. On the right-hand side are the three dummy variables capturing interviewer-respondent dyads. The reference category is the male-male dyad. Country fixed effects in each model hold country-specific confounders such as national political culture constant (Johnson and Braun 2016). Model 1 tests our main effects and Model 2 adds controls.

We use ordered logistic regression models.^14^ Given the ordinal nature of our dependent variable, we deem this analytical choice in line with theory. To interpret the logistic regression coefficients, we create marginal effects plots that display the predictive margins of interviewer gender on responses. In more detail, we create two marginal effects plots for each model. The first plot shows the predicted average marginal effect for men, split by interviewer gender. The second plot shows the predicted average marginal effect for women, split by interviewer gender.

To examine hypotheses 2a and 2b, we run an additional model where we interact two indicator variables capturing the gender of the interviewer (0 = female, 1 = male) and the gender of respondent (0 = female, 1 = male). This model performs a significance test of whether the gender of interviewer effect is stronger for men or for women. We also display these effects graphically via a conditional marginal effects plot.

To investigate hypotheses 3 to 5, we generate interactive models. We start by interacting the interviewer-respondent dyads and our measure of spousal presence. We then create models that control for the age difference between interviewers and respondents and proceed to interact the binary version of the 15-year age difference between the interviewer and respondent. In an additional model we also interact our dummies of interviewer-respondent dyads with respondent’s education. Similar to our main models, we also create marginal effects plots to graphically display these relationships.

## Results

Our analysis provides clear results. We find that the gender of the interviewer affects men’s and women’s responses to the gendered leadership item. Table 2 shows that regardless of interviewer gender, men are more supportive than women of the statement that men are better suited for political leadership, with women stating more support for gender equality in political leadership. Yet, the gender of the interviewer matters as well. Chi-square tests of independence, reported in table 2, show that there is a significant difference in answers across male and female interviewers to the gendered leadership item: respondents are less likely to agree with women’s suitability as leaders when interviewed by a man, and more likely to do so when interviewed by a woman. These results offer general support for hypothesis 1.

**Table 2.** Response distributions for the gendered leadership item by respondent and interviewer gender

	Male respondents		
	Male interviewer	Female interviewer	Difference
	%	%	%
Very strongly agree that “Men make better political leaders than women”	27.1	16.1	11
Agree that “Men make better political leaders than women”	15.7	11.9	3.8
Agree with neither	1.5	1.5	0.1
Agree that “Women should have the same chance of being elected to political office as men”	24.4	24.8	−0.4
Very strongly agree that “Women should have the same chance of being elected to political office as men”	31.3	45.8	−14.4

Total	100.0	100.0	
*X^2^* (4, *N *=* *26,566) = 349.85, *p* = 0.000			

	Female respondents		
	
	Male interviewer	Female interviewer	Difference
	%	%	%

Very strongly agree that “Men make better political leaders than women”	21.4	12.8	8.6
Agree that “Men make better political leaders than women”	11.9	9.2	2.7
Agree with neither	1.7	1.2	0.4
Agree that “Women should have the same chance of being elected to political office as men”	23.6	23.4	0.2
Very strongly agree that “Women should have the same chance of being elected to political office as men”	41.5	53.3	−11.8

Total	100.0	100.0	
*X^2^* (4, *N *=* *26,809) = 182.52, *p* = 0.000			

**Figure 1.**
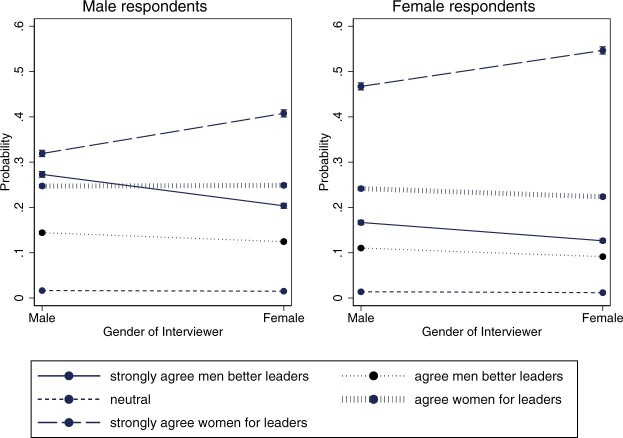
**Predicted average marginal effect of interviewer gender on responses to the gendered leadership item: by respondent gender**.

Our multivariate regression models (i.e., Models 1 and 2 in table 3) confirm this finding. For example, holding everything else constant in the model, men have an approximately 10-percentage-point higher chance of strongly agreeing with the statement that women make as good political leaders as men when interviewed by a woman than when interviewed by a man (the predicted probability to give this answer increases from 30 to 40 percent, as shown in figure 1). Women have an approximately 8-percentage-point higher chance of strongly agreeing with the statement that women are equally suitable for political leadership as men when the interviewer is female compared to when the interviewer is male.

**Table 3.** Multivariate ordinal logistic regression model of the gender of interviewer effects on responses to the gendered leadership item (ordered log odds regression coefficients, standard errors in parentheses)

	Model 1			Model 2			Model 3		
	Log odds	SE	*p*	Log odds	SE	*p*	Log odds	SE	*p*
Respondent male/interviewer male	Ref. cat.			Ref. cat.			Ref. cat.		
Respondent male/interviewer female	0.636	(0.023)	0.000	0.657	(0.023)	0.000	0.694	(0.028)	0.000
Respondent female/interviewer male	0.404	(0.023)	0.000	0.402	(0.023)	0.000	0.473	(0.028)	0.000
Respondent female/interviewer female	0.966	(0.023)	0.000	0.993	(0.024)	0.000	1.011	(0.029)	0.000
No one present				Ref. cat.			Ref. cat.		
Spouse present				0.028	(0.032)	0.392	0.249	(0.060)	0.000
Others present				0.018	(0.020)	0.360	0.084	(0.040)	0.037
Spouse present * resp. male/interv. female							−0.232	(0.090)	0.010
Spouse present * resp. female/interv. male							−0.418	(0.084)	0.000
Spouse present * resp. female/interv. female							−0.240	(0.092)	0.009
Others present * resp. male/interv. female							−0.084	(0.054)	0.118
Others present * resp. female/interv. male							−0.165	(0.056)	0.003
Others present * resp. female/interv. female							−0.023	(0.054)	0.671
Age difference (15 years)				0.072	(0.041)	0.080	0.072	(0.041)	0.081
No formal education				Ref. cat.			Ref. cat.		
Primary education				0.080	(0.027)	0.003	0.080	(0.027)	0.003
Secondary education				0.285	(0.027)	0.000	0.284	(0.027)	0.000
Post-secondary education				0.458	(0.033)	0.000	0.456	(0.032)	0.000
Christian				Ref. cat.			Ref. cat.		
Muslim				−0.223	(0.028)	0.000	−0.223	(0.028)	0.000
Other religion				−0.142	(0.030)	0.000	−0.142	(0.030)	0.000
Urban				0.073	(0.018)	0.000	0.073	(0.018)	0.000
Country fixed effects	Yes			Yes			Yes		
Cut-off point 1	0.243	(0.055)		0.388	(0.068)		0.414	(0.069)	
Cut-off point 2	0.942	(0.055)		1.067	(0.068)		1.095	(0.069)	
Cut-off point 3	1.015	(0.055)		1.139	(0.068)		1.166	(0.069)	
Cut-off point 4	2.092	(0.056)		2.216	(0.069)		2.244	(0.069)	
Log likelihood	−69,008.195			−66,230.739			−66,214.95		
LR chi2	5,227.54			5,257.99			5,289.57		
Prob > chi2	0.000			0.000			0.000		
Pseudo R^2^	0.04			0.04			0.04		
N	53,375			51,624			51,624		

**Figure 2.**
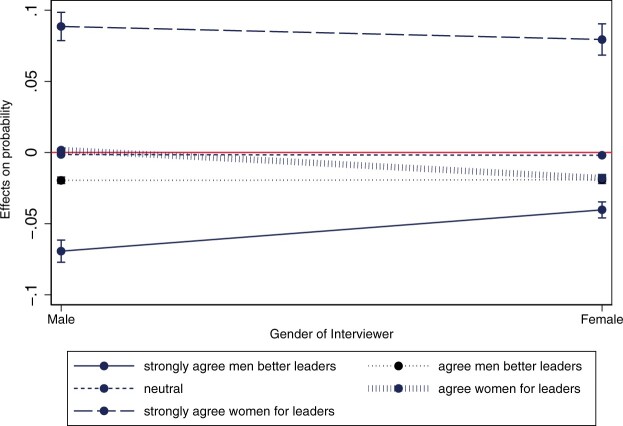
**Conditional marginal effect of the interviewer’s gender on men and women’s responses to the gendered leadership item**.

Table 4 illustrates that this interviewer effect is larger for male respondents. The negative interaction (*p* = 0.043 in the full model 2) between the gender of the interviewer and the gender of the respondent indicates that compared to women, men have a statistically higher likelihood of being affected by the gender of the interviewer. Figure 2 further displays that this effect is particularly strong for the first response category (i.e., those that “strongly agree that men make better leaders than women”). For the other response categories, this influence is smaller and there is an overlap in confidence intervals. We therefore find support for hypothesis 2b, albeit limited, which speaks against the proposition that women are more prone to engage in socially desirable responding when answering sensitive items (Lueptow et al. 1990).

**Table 4.** Multivariate ordinal logistic regression model assessing whether the gendered interviewer effect is stronger for men or for women (ordered log odds regression coefficients, standard errors in parentheses)

	Model 1			Model 2		
	Log odds	SE	*p*	Log odds	SE	*p*
Respondent gender (male)	0.636	(0.022)	0.000	0.657	(0.023)	0.000
Interviewer gender (male)	0.404	(0.023)	0.000	0.402	(0.023)	0.000
Respondent gender * interviewer gender	−0.074	(0.032)	0.022	−0.067	(0.033)	0.043
No one present				Ref. cat.		
Spouse present				0.028	(0.032)	0.392
Others present				0.018	(0.020)	0.360
Age difference (15 years)				0.072	(0.041)	0.080
No formal education				Ref. cat.		
Primary education				0.080	(0.027)	0.003
Secondary education				0.285	(0.027)	0.000
Post-secondary education				0.458	(0.032)	0.000
Christian				Ref. cat.		
Muslim				−0.223	(0.028)	0.000
Other religion				−0.142	(0.030)	0.000
Urban				0.073	(0.018)	0.000
Country fixed effects	Yes			Yes		
Cut-off point 1	0.243	(0.055)		0.388	(0.068)	
Cut-off point 2	0.942	(0.055)		1.067	(0.068)	
Cut-off point 3	1.014	(0.055)		1.139	(0.068)	
Cut-off point 4	2.092	(0.056)		2.216	(0.069)	
Log likelihood	−69,008.195			−66,230.739		
LR chi2	5,227.54			5,257.99		
Prob > chi2	0.000			0.000		
Pseudo R^2^	0.04			0.04		
N	53,375			51,624		

**Figure 3.**
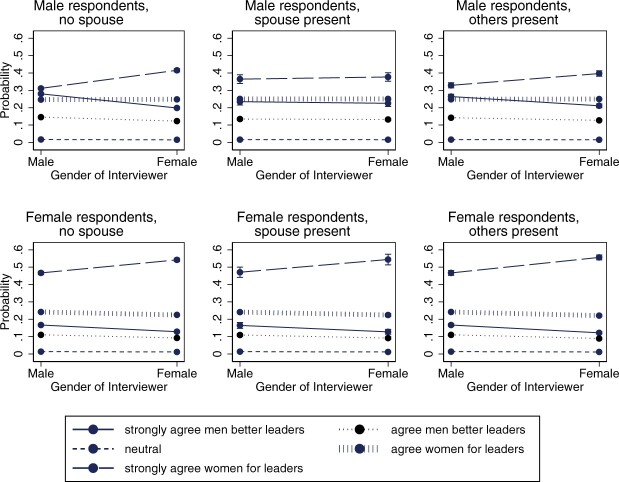
**Predicted average marginal effect of interviewer gender on responses to the gendered leadership item by respondent gender and spousal presence**.

The interactive model that explores the role of spousal presence (Model 3, table 3) illustrates that male respondents primarily seem to react differently to male and female interviewers if their wife is absent (see figure 3). This could be an indication that men are reluctant to show their traditional attitudes about women in politics (a preference for male leadership) in the presence of their wives. In contrast, there are no significant interactive effects for women; that is, women seem to alter their responses to interviewers regardless of whether their husbands are present or not. Our hypothesis 3 is therefore only partially supported.

**Figure 4.**
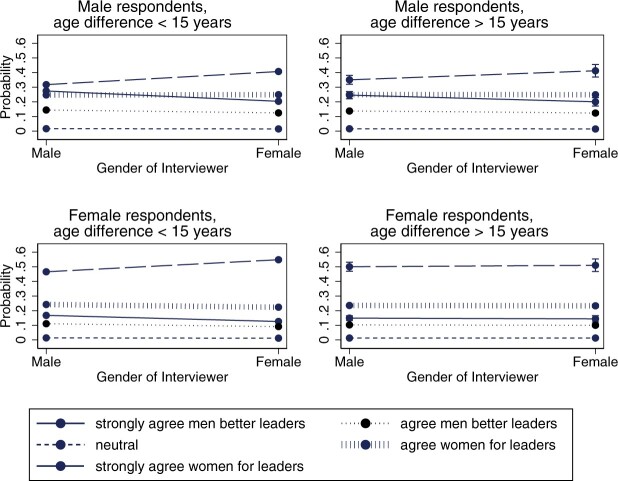
**Predicted average marginal effect of interviewer gender on responses to the gendered leadership item by respondent gender and the role of interviewer-respondent age difference**.

We find limited support for hypothesis 4. When we include the interactive terms capturing the 15-year age differences, we see that the effect of interviewer gender disappears among female respondents (see model 1 in table 5 and figure 4). Women give more progressive responses to female interviewers who are less than 15 years older than them but not to those at least 15 years older than them. This suggests to us that (relatively younger) women adapt their answers in the presence of older female interviewers. Yet, this finding comes with the caveat that the share of dyads where the interviewer is this much older and female is small (about 4 percent of our dyads; see table 1).^15^Figure 4 further illustrates that there are no significant interactive effects for men. In Supplementary Material table Sm10, we show that an age difference of five years and more (model Sm10a) and 10 years and more (model Sm10b) produces no significant interactions.

**Table 5.** Multivariate ordinal logistic regression models of gender of interviewer effects on responses to the gendered leadership item: age difference interactions and education interactions (ordered log odds regression coefficients, standard errors in parentheses)

	Model 1			Model 2		
	Log odds	SE	*p*	Log odds	SE	*p*
Respondent male/interviewer male	Ref. cat.			Ref. cat.		
Respondent male/interviewer female	0.657	(0.024)	0.000	0.625	(0.052)	0.000
Respondent female/interviewer male	0.408	(0.024)	0.000	0.686	(0.060)	0.000
Respondent female/interviewer female	1.004	(0.024)	0.000	1.213	(0.055)	0.000
No one present	Ref. cat.			Ref. cat.		
Spouse present	0.028	(0.032)	0.389	0.030	(0.032)	0.335
Others present	0.018	(0.020)	0.361	0.020	(0.020)	0.312
Age difference (15 years)	0.154	(0.073)	0.036	0.064	(0.041)	0.122
Age diff. (15) * resp. male/interv. female	−0.008	(0.100)	0.936			
Age diff. (15) * resp. female/interv. male	−0.132	(0.120)	0.272			
Age diff. (15) * resp. female/interv. female	−0.314	(0.118)	0.008			
No formal education	Ref. cat.			Ref. cat.		
Primary education	0.080	(0.027)	0.003	0.222	(0.051)	0.000
Secondary education	0.285	(0.027)	0.000	0.367	(0.049)	0.000
Post-secondary education	0.457	(0.032)	0.000	0.629	(0.057)	0.000
Primary education * resp. male/interv. Female				−0.049	(0.067)	0.468
Primary education * resp. female/interv. male				−0.303	(0.074)	0.000
Primary education * resp. female/interv. female				−0.278	(0.069)	0.000
Secondary education * resp. male/interv. female				0.101	(0.064)	0.118
Secondary education * resp. female/interv. male				−0.269	(0.070)	0.000
Secondary education * resp. female/interv. Female				−0.224	(0.067)	0.001
Post-second. education * resp. male/interv. female				0.175	(0.079)	0.027
Post-second. education * resp. female/interv. Male				−0.515	(0.080)	0.000
Post-second. education * resp. emale/interv. Female				−0.377	(0.081)	0.000
Christian	Ref. cat.			Ref. cat.		
Muslim	−0.223	(0.028)	0.000	−0.219	(0.028)	0.000
Other religion	−0.142	(0.030)	0.000	−0.139	(0.030)	0.000
Urban	0.073	(0.018)	0.000	0.074	(0.018)	0.000
Country fixed effects	Yes			Yes		
Cut-off point 1	.397	(0.068)		0.496	(0.075)	
Cut-off point 2	1.077	(0.068)		1.176	(0.075)	
Cut-off point 3	1.149	(0.068)		1.248	(0.075)	
Cut-off point 4	2.226	(0.069)		2.327	(0.076)	
Log likelihood	−66,226.345			−66,179.328		
LR chi2	5,266.78			5,360.81		
Prob > chi2	0.000			0.000		
Pseudo R-squared	0.04			0.04		
N	51,624			51,624		

**Figure 5.**
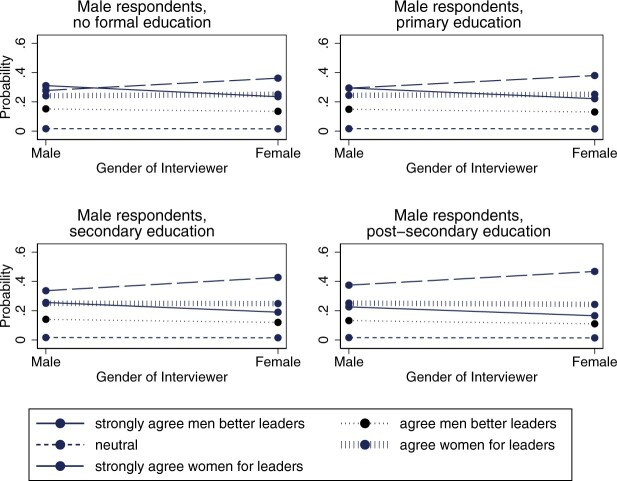
**Predicted average marginal effect of interviewer gender on responses to the gendered leadership item by education: male respondents**.

**Figure 6.**
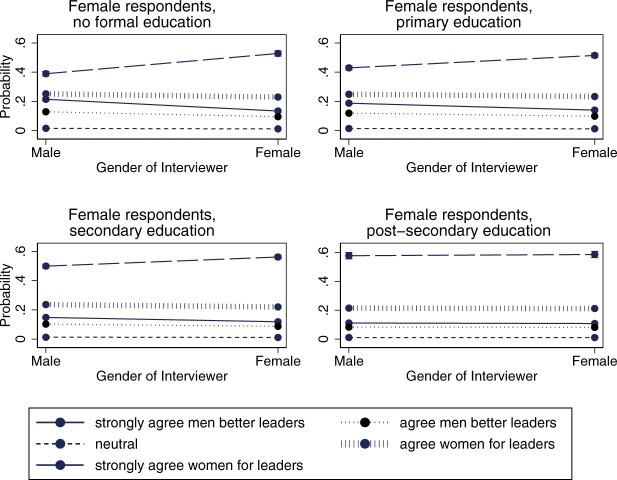
**Predicted average marginal effect of interviewer gender on responses to the gendered leadership item by education: female respondents**.

There is also an interactive effect between the interviewer-respondent dyads and respondents’ levels of education (model 2 in table 5 and figures 5 and 6). In support of hypothesis 5, we find that the effect of the gender of the interviewer is strongest for individuals with low education, and then levels off a bit more for each education level. We find support for this among both female and male respondents.

## Robustness Checks

We run two additional models with the same variable specification but now using generalized ordered logistic regressions and multinomial logistic regressions, because our ordered logistic regression analysis violates the parallel line assumption (Williams 2016). These two models (Supplementary Materia tables Sm11 and Sm12l) confirm our main results. We then show, using again the ordered logistic regression models, that our results are insensitive to different operationalizations of the dependent variable. For example, if we disregard the strength of opinion and reduce our dependent variable to three categories instead of five, results are consistent. The variables capturing gender of interviewer effects are all still significant (*p*<0.000) and the size of the coefficients and their directions are more or less unchanged (Supplemental Material table Sm3). The same applies if we run our main model on split samples of men and women (Supplementary Material tables Sm14 and Sm15).

Additional specifications indicate that the gender of interviewer effects are a rather consistent feature in the national surveys within the Afrobarometer project. Analyzing the main model for each of the 36 countries, we find that they persist in a majority: for 19 countries, all three interviewer-respondent dyads are significant, and for 16 countries at least two of the dyads are significant. In one country, only one of the dyads was significant (Supplementary Material table Sm16 and figure Sm1).

The findings are robust regardless of whether we run our models with country-level-, regional level-, local-level, or even enumeration area (EA)-level fixed effects (Supplementary Material table Sm17).^16^ Furthermore, when comparing models where we cluster standard errors on EAs or on interviewers, there are no visible differences in results (see Supplemental Material table Sm18). The results are also stable when we control for the levels of development in the area a respondent lives in. When including the three control variables of a local presence of a sewage system or health facilities, as well as whether there are roadblocks by the military or police visible in the area, our main coefficients remain unchanged as well (Supplementary Material table Sm19).

Moreover, these findings are unchanged when we run our main models on data from Afrobarometer rounds five and seven (for these replication results, see Supplemental Material table Sm1). Finally, we test the boundary conditions of our findings; that is, do these effects apply merely to survey items having to do with women’s standing in society, or to non-gendered questions as well? We do this through two types of analyses. First, we get very similar gender of interviewer effects when we regress three items—attitudes to women’s right to divorce, their right to work, and the chances of a women becoming a president in a Muslim society—on our interviewer-respondent dyads, respectively (Supplementary Material table Sm20).^17^ Second, and as a contrast, we do not find any gender of interviewer effects, if we look at two gender neutral items as the dependent variable (i.e., respondents’ trust in the president and their assessment of the nation’s economy) (Supplemental Material table Sm21).

## Conclusions

This study makes three contributions. First, its scope is larger than previous research. Adding to prior single-country studies (see Flores-Macias and Lawson 2008; Benstead 2014a), we present the first multinational study that looks at gender of interviewer effects holistically. We demonstrate that the interviewer’s gender influences men’s and women’s stated opinions about women’s suitability for political leadership, and these effects appear to be rather homogeneous across the African continent. Second, this study is among the first to empirically test whether the gender of interviewer effects are larger among men or among women. While the literature entertains both possibilities (Kane and Macaulay 1993; Huddy et al. 1997), we find larger effects among men when it comes to the response option that expresses the highest preference for male leaders. This finding further suggests that male respondents are more likely to engage in socially desirable reporting about women’s suitability for office. Third, we add a new, interesting finding to the literature: an age difference between the respondent and interviewer of at least 15 years eliminates the increase in women’s likelihood to state support for women in political leadership when they are interviewed by female compared to male interviewers. With the 15-year age gap, the gender of interviewer effect disappears among women. This suggests to us that women adapt their answers in the presence of older female interviewers. In addition, we also confirm some prior findings from the literature. As such, we endorse early analysis on spousal presence (see Zipp and Toth 2002). Our models illustrate that without their wives present men are more likely to voice preferences for male leadership than in their presence, irrespective of the gender of the interviewer. Finally, we find support for the proposition that gender of interviewer effects are larger among respondents with low education (see Campbell 1981; Blaydes and Gillum 2013).

Overall, our findings suggest to us that it may be inappropriate to estimate opinions about women’s suitability for political leadership within countries or to compare such estimates across countries using only responses from interviewer-administered surveys because such responses can be distorted by the interview conditions. Cross-country variation in data collection procedures such as the proportion of interviews conducted by male versus female interviewers, the proportion of men and women interviewed, age differences between interviewers and respondents, and so on, may distort country-level estimates and cross-country comparisons. In the data used here, for example, the share of interviews conducted by male interviewers ranged from 27 percent in South Africa to 75 percent in Cameroon (see Supplemental Material table Sm3), a difference that has potential to introduce considerable noise into comparisons between these two countries. Our results further suggest that analysts need to be able to account for characteristics of the interview itself (especially interviewer sex and age and who else was present during the interview), as well as the respondent characteristics of sex, age, and education, when estimating support for women’s leadership. However, in order to do so, analysts need access to information about interviewers and the interview context that is often unavailable in the world’s leading multinational surveys. Practically, we suggest that large survey programs, such as the World Values Survey and the European Values Survey, systematically report characteristics of the interviewer such as gender, as well as intersectional aspects of race, age and class, and religious symbols (see also Adida et al. 2016; Benstead 2014b). We also recommend that researchers then control for these items, when analyzing attitudinal questions, in particular those dealing with sensitive items such as gender equality.

## Data Availability Statement

REPLICATION DATA AND DOCUMENTATION are available at: https://dataverse.harvard.edu/dataset.xhtml?persistentId=doi:10.7910/DVN/VYNLON

## Supplementary Material

SUPPLEMENTARY MATERIAL may be found in the online version of this article: https://doi.org/10.1093/poq/nfac031.

## Supplementary Material

nfac031_Supplementary_Data

## Footnotes

1 Moreover, perceptions about whether interviewers represent the state may affect responses about support for democracy (Lau 2018) and government (Tannenberg 2022), especially in authoritarian settings (Calvo et al. 2019).

2 Much like the work of Krysan and Couper (2003), showing how white respondents are less likely to state negative racial attitudes in the presence of an African American interviewer.

3 This reasoning has similarities to theories of in-group behavior (cf. Tajfel et al. 1971), where (wo)men tend to identify with (fe)male interviewers because of shared group membership.

4 For the countries sampled in the Afrobarometer, the heterosexual norm is strong enough to make us believe that a reference to a spouse is a person of the opposite gender (i.e., it is likely that the presence of homophobia generally hinders LGBT people from safely stating to interviewers that they might have a same-sex spouse).

5 We refrain from using rounds five or seven, fielded two years prior and later, because they had only 34 countries. However, when replicated, our results remain largely the same with these additional rounds (see Supplementary Material table Sm1 for a basic model showing that the variables behave in similar ways across rounds and that main effects persist). The sample of Afrobarometer round six contains 53,415 respondents, the analytical samples for the regression models contain between 52,912 and 51,624 observations. For missing data, we engaged in listwise deletion. For sampling details, see https://afrobarometer.org/surveys-and-methods/sampling-principles. For figures on response rates, see https://afrobarometer.org/sites/default/files/data/afrobarometer_response_rates_round_2_to_7.xls. We used unweighted data to analyze all relationships between variables within the dataset.

6 While it is possible to investigate our questions on the Americas Barometer, the Arab Barometer, and the Asian Barometer, these projects do not ask the question about women’s leadership in the same way, lack several independent variables, and cover fewer countries.

7 Because of the binary categories used in the survey, we assume that people are sorted in two gender (or sex) categories. For this purpose, we use the terms male/man and female/women interchangeably. Respondent’s gender is recorded in the sampling phase and interviewer’s gender is self-reported.

8 Male and female interviewers are generally not assigned to different type of areas or respondents, but there is a small difference in the distribution of characteristics such as respondents’ age, education levels, and religious denominations across all four types of interviewer/respondent dyads, as well as residence type (see Supplementary Material tables Sm4–Sm7). However, we show in our models (e.g., models 2–7 and models Sm1–Sm20) that including controls for area type or respondents’ characteristics does not alter the gender of interviewer effects.

10 Information about who is attending is recorded by the interviewer in the questionnaire, and the assumption we make here is that the interviewer asks whether a person attending is the respondent’s spouse. As the third category includes the three response options “children,” “a small crowd,”’ or a few others,” it is possible that a spouse is present in the latter two categories. The option “spouse only” is not included in this category.

11 In additional models, we test for smaller age differences between interviewer and respondents (i.e., we create dummy variables if the age difference is at least 5 years or more, and at least 10 years or more, respectively), as well as larger age differences (i.e., 20 years’ difference and more).

12 We display the mean responses to this question across the four dyads for all countries in the dataset (Supplemental Material table Sm9).

13 We use Stata 17.

14 The models present the ordered log-odds (logit) regression coefficients and the cut-off points (which display different levels of support of the latent variable used to differentiate various levels of the support for women as leaders). For each variable, we present the ordered log odds regression coefficient, the standard error, and the significance level (*p*-values). We also present information on the log-likelihood of the model, a likelihood ratio (LR) Chi squared test—indicating whether a model is significant—and the Pseudo R-squared value.

15
Model Sm10c, in the Supplementary Material, shows that the interaction effect is somewhat larger when the age difference is 20 years or more.

16 We accessed “geocoded” Afrobarometer data and created dummies for EAs (BenYishay et al. 2018).

17 These questions were posed in a subsample of five Arab countries and therefore are not viable as our main focus.

9 For a distribution per each country, including the “don’t know,” see Supplemental Material table Sm8.

## Notes

The authors thank Jane Mansbridge and Archon Fung for hosting Sundström as a Visiting Democracy Scholar at Harvard University, where this article was initiated. They thank Lena Wängnerud, Henrik Ekengren Oscarsson, and Johan Martinsson for organizing workshops at the University of Gothenburg, where drafts were presented. The authors benefited from comments by various colleagues, including Mattias Agerberg, Sofia Axelsson, Karen E. Ferree, Ellen Lust, Kristen Kao, Cem Mert Dalli, Marcus Tannenberg, Christian Welzel, and Pär Zetterberg. This work was supported by the Swedish Research Council [2019-03218 to A.S].

## References

[nfac031-B1] Adida Claire L. , Ferree Karen E., Posner Daniel N., Robinson Amanda L. 2016. “Who’s Asking? Interviewer Coethnicity Effects in African Survey Data.” Comparative Political Studies 49:1630–60.

[nfac031-B2] Afrobarometer. 2014. “Round 6 Survey Manual.” Compiled by the Afrobarometer, May.

[nfac031-B3] Aquilino William S. 1993. “Effects of Spouse Presence During the Interview on Survey Responses Concerning Marriage.” Public Opinion Quarterly 57:358–76.

[nfac031-B4] Becker Stan , Feyisetan Kale, Makinwa-Adebusoye Paulina. 1995. “The Effect of the Sex of Interviewers on the Quality of Data in a Nigerian Family Planning Questionnaire.” Studies in Family Planning 26:233–40. 7482680

[nfac031-B5] Benney Mark , Riesman David, Star Shirley A. 1956. “Age and Sex in the Interview.” American Journal of Sociology 62:143–52.

[nfac031-B6] Benstead J. Lindsay. 2014a. “Effects of Interviewer–Respondent Gender Interaction on Attitudes toward Women and Politics: Findings from Morocco.” International Journal of Public Opinion Research 26:369–83.

[nfac031-B7] Benstead J. Lindsay. 2014b. “Does Interviewer Religious Dress Affect Survey Responses? Evidence from Morocco.” Politics and Religion 7:734–60.

[nfac031-B8] Benstead J. Lindsay. 2018a. “Survey Research in the Arab World: Challenges and Opportunities.” PS: Political Science & Politics 51:535–42.

[nfac031-B9] Benstead J. Lindsay. 2018b. “Survey Research in the Arab World.” In The Oxford Handbook of Polling and Survey Methods, edited by Atkeson Lonna Rae, Michael Alvarez R., 220–38. Oxford: Oxford University Press.

[nfac031-B10] BenYishay Ariel , Rotberg Renee, Wells Jessica, Lv Zhonghui, Goodman Seth, Kovacevic Lidia, Runfola Dan. 2018. “Geocoding Afrobarometer: Methodology & Data Quality.” Virginia: AidData.

[nfac031-B11] Blaydes Lisa , Gillum Rachel M. 2013. “Religiosity-of-Interviewer Effects: Assessing the Impact of Veiled Enumerators on Survey Response in Egypt.” Politics and Religion 6:459–82.

[nfac031-B12] Bolzendahl Catherine I. , Myers Daniel J. 2004. “Feminist Attitudes and Support for Gender Equality: Opinion Change in Women and Men, 1974–1998.” Social Forces 83:759–89.

[nfac031-B13] Brenner Philip. 2017. “Toward a Social Psychology of Survey Methodology: An Application of the Approach and Directions for the Future.” Sociology Compass 11:e12491.

[nfac031-B14] Calvo Thomas , Razafindrakoto Mireille, Roubaud François. 2019. “Fear of the State in Governance Surveys? Empirical Evidence from African Countries.” World Development 123:104609. 10.1016/j.worlddev.2019.104609

[nfac031-B15] Campbell Bruce A. 1981. “Race-of-Interviewer Effects among Southern Adolescents.” Public Opinion Quarterly 45:231–44.

[nfac031-B16] Catania Joseph A. , Binson Diane, Canchola Jesse, Pollack Lance M., Huack Walter, Coates Thomas J. 1996. “Effects of Interviewer Gender, Interviewer Choice, and Item Wording on Responses to Questions Concerning Sexual Behavior.” Public Opinion Quarterly 60:345–75.

[nfac031-B17] Davis Rachel E. , Couper Mick P., Janz Nancy K., Caldwell Cleopatra H., Resnicow Ken. 2010. “Interviewer Effects in Public Health Surveys.” Health Education Research 25:14–26. 19762354 10.1093/her/cyp046 PMC2805402

[nfac031-B18] Dijkstra Wil. 1987. “Interviewing Style and Respondent Behavior: An Experimental Study of the Survey-Interview.” Sociological Methods & Research 16:309–34.

[nfac031-B19] Dykema Jennifer , Diloreto Kerryann, Price Jessica, White Eric, Schaeffer Nora. 2012. “ACASI Gender-of-Interviewer Voice Effects on Reports to Questions about Sensitive Behaviors among Young Adults.” Public Opinion Quarterly 76:311–25. 24991062 10.1093/poq/nfs021 PMC4079084

[nfac031-B20] Fisher Robert J. 1993. “Social Desirability Bias and the Validity of Indirect Questioning.” Journal of Consumer Research 20:303–15.

[nfac031-B21] Flores-Macias Francisco , Lawson Chappel. 2008. “Effects of Interviewer Gender on Survey Responses: Findings from a Household Survey in Mexico.” International Journal of Public Opinion Research 20:100–110.

[nfac031-B22] Frisone John D. , Galla John P., Jeffrey Linda R., Gaer Eleanor P. 1982. “Effect of Communicating Experimenter Attitudes on Subject Response to a Sex-Role Attitude Questionnaire.” Journal of Psychology 111:27–29.

[nfac031-B23] Galla John P. , Frisone John D., Jeffrey Linda R., Gaer Eleanor P. 1981. “Effect of Experimenter’s Gender on Responses to a Sex-Role Attitude Questionnaire.” Psychological Reports 49:935–40.

[nfac031-B24] Grenz Sabine. 2005. “Intersections of Sex and Power in Research on Prostitution: A Female Researcher Interviewing Male Heterosexual Clients.” Signs: Journal of Women in Culture and Society 30:2091–113.

[nfac031-B25] Groves Robert M. , Fultz Nancy H. 1985. “Gender Effects among Telephone Interviewers in a Survey of Economic Attitudes.” Sociological Methods & Research 14:31–52.

[nfac031-B26] Hartmann Petra. 1994. “Interviewing When the Spouse Is Present.” International Journal of Public Opinion Research 6:298–306.

[nfac031-B27] Hill Charles T. , Stull Donald E. 1987. “Gender and Self-Disclosure: Strategies for Exploring the Issues.” In Self-Disclosure: Theory, Research, and Therapy, edited by Valerian J. Derlega, John H. Berg, pp. 81–100. New York: Plenum Press.

[nfac031-B28] Huddy Leonie J. , Billig Joshua, Bracciodieta John, Hoeffler Lois, Moynihan Patrick J., Pugliani Patricia. 1997. “The Effect of Interviewer Gender on the Survey Response.” Political Behavior 19:197–220.

[nfac031-B29] Hyman Herbert H. , Cobb William J., Feldman Jacob J., Hart Clyde, Stember Charles H. 1954. Interviewing in Social Research. Chicago: University of Chicago Press.

[nfac031-B30] Inglehart Ronald F. , Norris Pippa. 2003. Rising Tide: Gender Equality and Cultural Change Around the World. Cambridge: Cambridge University Press.

[nfac031-B31] Johnson Timothy P. , Braun Michael. 2016. “Challenges of Comparative Survey Research.” In The SAGE Handbook of Survey Methodology, edited by Wolf Christof, Dominique Joye, Smith Tom W., Fu Yang-chih, 41–54. London: SAGE.

[nfac031-B32] Kane Emily W. , Macaulay Laura J. 1993. “Interviewer Gender and Gender Attitudes.” Public Opinion Quarterly 57:1–28.

[nfac031-B33] Krysan Maria , Couper Mick P. 2003. “Race in the Live and the Virtual Interview: Racial Deference, Social Desirability, and Activation Effects in Attitude Surveys.” Social Psychology Quarterly 66:364–83.

[nfac031-B34] Krysan Maria , Couper Mick P., Farley Reynolds, Forman Tyrone A. 2009. “Does Race Matter in Neighborhood Preferences? Results from a Video Experiment.” AJS; American Journal of Sociology 115:527–59. 20614764 10.1086/599248 PMC3704191

[nfac031-B35] Landis Judson R. , Sullivan Daryl, Sheley Joseph. 1973. “Feminist Attitudes as Related to Sex of the Interviewer.” Pacific Sociological Review 16:305–14.

[nfac031-B36] Lau Charles Q. 2018. “The Influence of Interviewer Characteristics on Support for Democracy and Political Engagement in Sub-Saharan Africa.” International Journal of Social Research Methodology 21:467–86.

[nfac031-B37] Liu Mingnan , Wang Yichen. 2016. “Interviewer Gender Effect on Acquiescent Response Style in 11 Asian Countries and Societies.” Field Methods 28:327–44.

[nfac031-B38] Lovenduski Joni , Norris Pippa. 1993. Gender and Party Politics. London: SAGE.

[nfac031-B39] Lueptow Lloyd B. , Moser Susan L., Pendleton Brian F. 1990. “Gender and Response Effects in Telephone Interviews about Gender Characteristics.” Sex Roles 22:29–42.

[nfac031-B40] Lupu Noam , Michelitch Kristin. 2018. “Advances in Survey Methods for the Developing World.” Annual Review of Political Science 21:195–214.

[nfac031-B41] Mneimneh Zeina N. , Elliott Michael R., Tourangeau Roger, Heeringa Steven G. 2018. “Cultural and Interviewer Effects on Interview Privacy: Individualism and National Wealth.” Cross-Cultural Research 52:496–523.

[nfac031-B42] Nederhof Anton J. 1985. “Methods of Coping with Social Desirability Bias: A Review.” European Journal of Social Psychology 15:263–80.

[nfac031-B43] Olson Kristen , Smyth Jolene D., Dykema Jennifer, Holbrook Allyson L., Kreuter Frauke, West Brady T. 2020. “The Past, Present, and Future of Research on Interviewer Effects.” In Interviewer Effects from a Total Survey Error Perspective, edited by Olson Kristen, Smyth Jolene D., Dykema Jennifer, Holbrook Allyson L., Kreuter Frauke, West Brady T., pp. 3–16. London: Chapman & Hall/CRC.

[nfac031-B44] Paulhus Delroy L. 2002. “Socially Desirable Responding: The Evolution of a Construct.” In The Role of Constructs in Psychological and Educational Measurement, edited by Braun Henry I., Jackson Douglas N., Wiley David E., pp. 49–69. Mahwah: Lawrence Erlbaum Associates.

[nfac031-B45] Pew Research Center. 2015. “The Whys and Hows of Generation Research.” https://www.pewresearch.org/politics/2015/09/03/the-whys-and-hows-of-generations-research/. Date accessed January 10, 2022.

[nfac031-B46] Pollner Melvin. 1998. “The Effects of Interviewer Gender in Mental Health Interviews.” Journal of Nervous & Mental Disorder 186:369–73. 10.1097/00005053-199806000-00008 9653422

[nfac031-B47] Schuman Howard , Converse Jean M. 1971. “The Effects of Black and White Interviewers on Black Responses.” Public Opinion Quarterly 35:44–68.

[nfac031-B48] Smith Tom. 1997. “The Impact of the Presence of Others on a Respondent’s Answers to Questions.” International Journal of Public Opinion Research 9:33–47.

[nfac031-B49] Stout Christopher T. , Kline Reuben. 2011. “‘I’m Not Voting for Her’: Polling Discrepancies and Female Candidates.” Political Behavior 33:479–503.

[nfac031-B50] Streb Matthew J. , Burrell Barbara, Frederick Brian, Genovese Michael A. 2008. “Social Desirability Effects and Support for a Female American President.” Public Opinion Quarterly 72:76–89.

[nfac031-B51] Sundström Aksel , Paxton Pamela, Wang Yi-Ting, Lindberg Staffan. 2017. “Women’s Political Empowerment: A New Global Index.” World Development 94:321–35. 10.1016/j.worlddev.2017.01.016 PMC11139198 38817881

[nfac031-B52] Tajfel Henri , Billig Michael G., Bundy Robert P., Flament Claude. 1971. “Social Categorization and Intergroup Behaviour.” European Journal of Social Psychology 1:149–78.

[nfac031-B53] Tannenberg Marcus. 2022. “The Autocratic Bias: Self-censorship of Regime Support.” Democratization 29:591–610.

[nfac031-B54] Tu Su-Hao , Liao Pei-Shan. 2007. “Social Distance, Respondent Cooperation and Item Nonresponse in Sex Survey.” Quality & Quantity 41:177–99.

[nfac031-B55] West Brady T. , Blom Annelies G. 2017. “Explaining Interviewer Effects: A Research Synthesis.” Journal of Survey Statistics and Methodology 5:smw024.

[nfac031-B56] Williams J. Allen. 1964. “Interviewer-Respondent Interaction: A Study of Bias in the Information Interview.” Sociometry 27:338–52.

[nfac031-B57] Williams Richard. 2016. “Understanding and Interpreting Generalized Ordered Logit Models.” Journal of Mathematical Sociology 40:7–20.

[nfac031-B58] Zipp John F. , Toth Joann. 2002. “She Said, He Said, They Said: The Impact of Spousal Presence in Survey Research.” Public Opinion Quarterly 66:177–208.

