# Supplementary material for: Measuring Support for Women’s Political Leadership: Gender of Interviewer Effects Among African Survey Respondents
Source: Public Opin Q. 2022 Sep 2;86(3):668–96. doi: 10.1093/poq/nfac031 (PMC9521196; doi:10.1093/poq/nfac031)
Supplement: nfac031_Supplementary_Data [file nfac031_supplementary_data.pdf]

Measuring support for women's political leadership:

Gender of interviewer effects among African survey respondents

Public Opinion Quarterly Vol 86(3)

Aksel Sundström

Associate Professor, the Quality of Government Institute, Department of Political Science, University of Gothenburg, PO Box 711 405 50, Gothenburg, Sweden

e-mail: aksel.sundstrom@pol.gu.se

Daniel Stockemer

Professor, School of Political Studies, Faculty of Social Sciences, 120 University Social Sciences, Building Room 7005, Ottawa, Ontario, Canada K1N 6N5

e-mail: daniel.stockemer@uottawa.ca

Supplemental materials

Table Sm1. Multivariate ordinal logistic regression model of the gender of interviewer effects on assessments of whether men make better political leaders than women, using Rounds 5 and 7 (ordered log odds regression coefficients, standard errors in parentheses)

	Model Sm1a (Round 5)		Model Sm1b (Round 7)	
		p		p
Respondent male/ interviewer male	Ref. cat.		Ref. cat.	
Respondent male/ interviewer female	.573 (.023)	.000	.663 (.025)	.000
Respondent female/ interviewer male	.342 (.024)	.000	.420 (.026)	.000
Respondent female/ interviewer female	.791 (.024)	.000	.952 (.027)	.000
No one present	Ref. cat		Ref. cat	
Spouse present	-.069 (.033)	.034	-.110 (.038)	.004
Others present	-.047 (.020)	.021	-.076 (.021)	.000
Age difference (15 years)	.103 (.043)	.017	-.016 (.042)	.703
No formal education	Ref. cat		Ref. cat	
Primary education	.153 (.026)	.000	.207 (.029)	.000
Secondary education	.394 (.026)	.000	.503 (.030)	.000
Post-secondary education	.662 (.033)	.000	.702 (.036)	.000
Christian	Ref. cat		Ref. cat	
Muslim	-.128 (.027)	.000	-.206 (.030)	.000
Other religion	.003 (.028)	.910	-.051 (.031)	.100
Urban	.095 (.019)	.000	-.021 (.020)	.306
Country fixed effects	Yes		Yes	
Cut-off point 1	-1.390 (.060)		-1.253 (.063)	
Cut-off point 2	-.571 (.060)		-.660 (.063)	
Cut-off point 3	-.497 (.060)		-.581 (.063)	
Cut-off point 4	.758 (.060)		.324 (.063)	
Log likelihood	-64816.674		-54169.892	
LR chi2	5259.39		3804.40	
Prob > chi2	.000		.000	
Pseudo Rsquared	.04		.03	
N	50374		44847	

Supplemental materials Sm2. Interviewer teams and assignment of male/female interviewers

The Afrobarometer protocol guides that the survey is completed in the same way in all partner countries and the organization trains the national team in its implementation. Because a representative of the Afrobarometer is present during training of interviewer teams, this safeguard that the protocol is rather uniform across countries. When fielded, interviewers move in teams consisting of one field supervisor and four interviewers (Afrobarometer, 2014, p. 6). These interviewer-teams consists of both men and women. Besides being a goal in the survey manual – stating that "interviewer teams should be comprised of a minimum of 40% women," (p.10) – we corroborated that this is the case in practice.

First, we interviewed the Deputy Director of Surveys of the Afrobarometer organization (Ms. Anyway Chingwete, Feb 25th, 2021). From this interview it was made clear that teams always consist of both men and women. Although the aim of the overall share of women is not always met for a country as a whole, the teams are not gender-separated (i.e., they do not use male-only, or female-only teams). It was made clear that if there are concerns for security in an area (e.g. if the presence of violence makes survey conduct too dangerous), then the supervisor makes the decision to withdraw from the entire enumeration area (EA). In this sense, the full team moves to another EA and not only some of the interviewers. Because teams are gender-mixed, withdrawing form an insecure EA will not disproportionally affect which areas are surveyed by male or female interviewers.

Second, we contacted national partner firms in February 2021 to ask them about the composition of their interviewer teams. In total, we communicated with spokespersons of 15 partners firms per email during February and March 2021.[1] All of them depict a situation where teams are indeed gender-mixed. The firms described that while an even gender-balance is not always achieved in each team, they do not use teams consisting of only one gender. As explained in detail by partner firms in Eswatini and in Kenya, this is partly because of security reasons, to make sure female interviewers are working in the presence of men as well.

1

Supplemental materials Table Sm2

**Country**	**Afrobarometer national partner firm**
Angola	Ovilongwa
Botswana	Star Awards
Burkina Faso	Centre pour la Gouvernance Démocratique (CGD)
Burundi	Groupe de Recherche et d'Appui aux Initiatives Democratiques (GRADIS)
Cameroon	le Groupe Cible
Cote D'Ivoire	Centre de Recherche et de Formation sur le Développement Intégré (CREFDI)
Eswatini	ActivQuest
Gabon	Center of Studies and Research on Geosciences Politics and Prospective (CERGEP)
Kenya	Institute for Development Studies (IDS)
Morocco	Global Survey and Consulting (GSC)
Nigeria	NOIPolls
South Africa	Institute for Justice and Reconciliation
Sudan	Sudan Polling Statistics Center
Tanzania	Policy Research for Development (REPOA)
Togo	Centre de Recherche et de Sondage d'Opinions (CROP)

Table Sm3. Distributions of interviewer gender and the presence of third parties present, per country

Countries	Number male interviewers	Number female interviewers	Share of interviews with male interviewers	Share of interviews with female interviewers	Share no one else present	Share with spouse as only third party present	Share with others present
Algeria	14	14	50	50	39.08	13.75	47.17
Benin	15	13	54	46	89.83	2.33	7.83
Botswana	14	14	50	50	64.94	5.59	29.47
Burkina Faso	13	11	55	45	76.83	3.83	19.33
Burundi	14	14	50	50	90.56	1.25	8.19
Cameroon	18	6	74.96	25.04	64.71	8.84	26.45
Cape Verde	14	12	50.5	49.5	66.73	5.41	27.86
Cote d’Ivoir	14	10	58.38	41.62	85.24	2.09	12.68
Egypt	39	29	50.33	49.67	53.33	10.46	36.20
Eswatini	14	18	44.42	55.58	68.58	3.75	27.67
Gabon	15	13	53.42	46.58	51.59	6.84	41.57
Ghana	28	23	51.83	48.17	57.94	6.52	35.55
Guinea	14	10	58.17	41.83	83.83	5.42	10.75
Kenya	19	16	52.77	47.23	53.71	8.10	38.19
Lesotho	6	6	50	50	68.75	7.67	23.58
Liberia	17	15	51.88	48.12	53.81	8.38	37.80
Madagascar	19	15	54.33	45.67	77.33	9.42	13.25
Malawi	27	23	52.79	47.21	78.08	4.79	17.12
Mali	15	15	49.33	50.67	97.58	0.75	1.67
Mauritius	18	13	58.25	41.75	70.33	16.33	13.33
Morocco	10	10	50.67	49.33	51.25	10.92	37.83
Mozambique	28	25	53.21	46.79	43.50	11.75	44.75
Namibia	8	9	39.08	60.92	73.08	5.58	21.33
Niger	11	11	52.33	47.67	74.06	8.76	17.18
Nigeria	15	15	51.21	48.79	60.90	7.46	31.64
San Tome	8	7	50.59	49.41	79.93	2.60	17.46
Senegal	12	8	60.67	39.33	69.37	6.68	23.96
Sierra Leone	10	6	62.30	37.70	89.24	2.77	7.98
South Africa	23	49	27.45	72.55	65.44	9.71	24.85
Sudan	11	17	38.75	61.25	56.42	12.08	31.50
Tanzania	10	15	37.59	62.41	75.23	4.86	19.91
Togo	14	16	45.42	54.58	74.73	2.75	22.52
Tunisia	14	11	54.08	45.92	40.58	11.00	48.42
Uganda	15	15	51.00	49.00	60.29	8.62	31.08
Zambia	21	17	53.04	46.96	52.26	9.95	37.79
Zimbabwe	23	25	49.25	50.75	55.99	13.02	31.00

Figures refer to Afrobarometer Round 6.

Table Sm4. Distribution of interviewer-respondent ratio by urban/ rural (numbers, percentages in parentheses)

	Urban	Rural
Respondent male/ interviewer male	8238 (60.1)	5470 (39.9)
Respondent male/ interviewer female	8116 (59.91)	5431 (40.09)
Respondent female/ interviewer male	7279 (55.59)	5814 (44.41)
Respondent female/ interviewer female	7613 (56.03)	5974 (43.97)
Average share	57.93	42.07

Chi-square tests of independence showed that there was a significant association between the respondent-interviewer dyads and location of respondents, X2 (3, N = 53935) = 97.58, p = .000

Table Sm5. Distribution of interviewer-respondent ratio by age (means)

	Age, years
Respondent male/ interviewer male	38.33
Respondent male/ interviewer female	35.85
Respondent female/ interviewer male	38.44
Respondent female/ interviewer female	36.46
Average	37.27

An one-way Anova tests showed that there was a significant association between the respondent-interviewer dyads and mean age of respondents (F(3,3) = 109.6, p = .000).

Table Sm6. Distribution of interviewer respondent ratio by education (numbers, percentages in parentheses)

	No formal	Primary	Secondary	Post-secondary
Respondent male/ interviewer male	2351 (17.19)	3760 (27.49)	5166 (37.77)	2401 (17.55)
Respondent male/ interviewer female	3151 (23.33)	4092 (30.30)	4466 (33.07)	1796 (13.3)
Respondent female/ interviewer male	1923 (14.72)	3553 (27.21)	5135 (39.32)	2449 (18.75)
Respondent female/ interviewer female	2798 (20.67)	4169 (30.80)	4707 (34.77)	1863 (13.76)
Average share	19.01	28.96	36.21	15.82

Chi-square tests of independence showed that there was a significant association between the respondent-interviewer dyads and education levels of respondents, X2 (3, N = 53780) = 626.6262, p = .000

Table Sm7. Distribution of interviewer respondent ratio by religion (numbers, percentages in parentheses)

	Christian	Muslim	Other
Respondent male/ interviewer male	7622 (56.99)	4293 (32.1)	1459 (10.91)
Respondent male/ interviewer female	8094 (61.21)	3997 (30.23)	1133 (8.57)
Respondent female/ interviewer male	7792 (61.08)	3577 (28.04)	1389 (10.89)
Respondent female/ interviewer female	8365 (63.07)	3756 (28.32)	1141 (8.6)
Average share	60.57	29.69	9.73

Chi-square tests of independence showed that there was a significant association between between the respondent-interviewer dyads and religious denomination of respondents, X2 (3, N = 52618) = 165.0261, p = .000

Table Sm8. Distributions of the responses to question on whether men or women make better leaders, per country

Countries	Share ‘Strongly agree with statement one’	Share ‘Agree with statement one’	Share ‘Agree with neither’	Share ‘Agree with statement two’	Share ‘Strongly agree with statement two’	‘Don’t know’
Algeria	41.92	17.25	3.42	19.75	15.67	2.00
Benin	14.08	12.92	0.17	28.92	43.67	0.25
Botswana	6.50	8.58	0.92	28.75	54.92	0.33
Burkina Faso	29.67	8.83	1.25	20.00	39.75	0.50
Burundi	12.33	13.92	0.58	25.17	47.92	0.08
Cameroon	14.04	15.74	0.25	29.10	39.09	1.78
Cape Verde	2.83	4.42	0.58	38.83	52.83	0.50
Cote d’Ivoir	8.84	11.34	0.17	39.87	39.70	0.08
Egypt	21.20	30.13	2.84	26.96	13.61	5.26
Eswatini	9.83	11.17	0.58	28.08	50.08	0.25
Gabon	6.18	6.51	0.42	34.64	52.17	0.08
Ghana	20.68	8.21	1.29	16.55	52.31	0.96
Guinea	25.17	13.08	0.42	18.83	42.25	0.25
Kenya	12.47	9.09	1.88	19.94	56.28	0.33
Lesotho	34.58	4.33	1.83	12.50	45.50	1.25
Liberia	27.80	14.19	0.42	29.30	27.96	0.33
Madagascar	19.33	19.42	0.42	29.50	31.33	0
Malawi	33.54	3.25	1.50	4.50	56.79	0.42
Mali	32.58	8.58	1.83	11.17	45.83	0
Mauritius	5.25	14.67	0.92	39.67	38.92	0.58
Morocco	14.92	16.67	1.58	31.42	33.17	2.25
Mozambique	11.12	15.46	2.04	30.08	36.25	5.04
Namibia	8.00	12.42	0.33	39.50	39.75	0
Niger	36.50	18.25	0.33	14.33	30.42	0.17
Nigeria	23.88	24.09	2.00	23.97	24.89	1.17
San Tome	11.06	15.42	4.61	30.43	34.95	3.52
Senegal	25.67	7.67	0.92	12.08	53.25	0.42
Sierra Leone	28.80	7.89	4.37	13.85	42.74	2.35
South Africa	13.26	12.43	2.93	22.18	48.37	0.84
Sudan	30.83	23.67	2.00	29.42	12.58	1.50
Tanzania	15.60	15.01	0.38	30.57	37.57	0.88
Togo	5.58	6.00	0.83	28.33	59.17	0.08
Tunisia	27.83	8.25	1.08	26.25	35.75	0.83
Uganda	19.01	5.50	2.00	16.13	57.15	0.21
Zambia	18.86	8.26	0.83	25.29	46.08	0.67
Zimbabwe	21.25	8.54	1.62	15.88	52.21	0.50

Figures refer to Afrobarometer Round 6.

Table Sm9. Average assessments across countries: Gendered-respondent effects on individuals' assessment of whether men or women make better leaders

Countries	Respondent male/ interviewer male (mean responses)	Respondent male/ interviewer female (mean responses)	Respondent female/ interviewer male (mean responses)	Respondent female/ interviewer female (mean responses)
Algeria	1.02	1.00	1.95	2.00
Benin	2.19	2.74	2.91	3.25
Botswana	3.00	3.13	3.20	3.36
Burkina Faso	1.75	2.66	2.18	2.84
Burundi	2.37	3.00	2.75	3.18
Cameroon	2.41	2.71	2.63	3.37
Cape Verde	3.01	3.42	3.44	3.54
Cote d'Ivoir	2.42	2.80	3.23	3.22
Egypt	1.41	1.51	2.02	2.29
Eswatini	2.37	2.96	2.99	3.46
Gabon	3.03	3.12	3.26	3.42
Ghana	2.08	2.78	2.88	3.19
Guinea	1.84	2.93	2.23	2.98
Kenya	2.68	2.77	3.23	3.28
Lesotho	1.96	1.84	2.73	2.68
Liberia	1.87	2.13	2.38	2.24
Madagascar	1.93	2.13	2.54	2.80
Malawi	2.23	2.75	2.23	2.77
Mali	1.84	2.42	2.36	2.53
Mauritius	2.59	2.48	3.29	3.33
Morroco	2.00	1.87	3.14	3.09
Mozambique	2.71	2.54	2.85	2.59
Namibia	2.29	2.91	3.11	3.16
Niger	1.25	2.18	1.56	2.45
Nigeria	1.74	1.88	2.22	2.26
San Tome	2.22	3.07	2.31	3.04
Senegal	2.01	2.24	2.98	3.27
Sierra Leone	1.66	2.76	2.32	3.11
South Africa	2.45	2.44	3.13	3.17
Sudan	1.47	1.45	1.80	1.98
Tanzania	2.07	2.37	2.93	2.95
Togo	3.02	3.18	3.35	3.60
Tunisia	1.88	2.68	2.20	2.73
Uganda	2.35	2.82	3.01	3.31
Zambia	2.54	2.65	2.82	2.88
Zimbabwe	2.09	2.45	3.00	3.24

Figures refer to Afrobarometer Round 6. The variable ranges from 0 (agree very strongly with the statement that men make better leaders) to 4 (agree very strongly with the statement that women are equally good leaders), with a neutral mid-category.

Table Sm10. Multivariate ordinal logistic regression models of gender of interviewer effects on whether men make better political leaders: age differences, alternative specifications (ordered log odds regression coefficients, standard errors in parentheses)

	Model Sm10a		Model Sm10b		Model Sm10c	
		p		p		p
Respondent male/ interviewer male	Ref. cat.		Ref. cat.		Ref. cat.	
Respondent male/ interviewer female	.662 (.026)	.000	.657 (.025)	.000	.653 (.023)	.000
Respondent female/ interviewer male	.403 (.026)	.000	.406 (.024)	.000	.402 (.023)	.000
Respondent female/ interviewer female	1.003 (.027)	.000	1.002 (.025)	.000	1.000 (.024)	.000
No one present	Ref. cat		Ref. cat		Ref. cat	
Spouse present	.027 (.032)	.410	.027 (.032)	.395	.028 (.032)	.385
Others present	.018 (.020)	.362	.018 (.020)	.357	.018 (.018)	.370
Age difference (5 years)	.023 (.039)	.550				
Age diff. (5) * resp. male/ interv. Female	-.017 (.053)	.755				
Age diff. (5) * resp. female/ interv. Male	-.009 (.058)	.877				
Age diff. (5) * resp. female/ interv. Female	-.056 (.058)	.339				
Age difference (10 years)			.069 (.052)	.180		
Age diff. (10) * resp. male/ interv. Female			-.007 (.070)	.918		
Age diff. (10) * resp. female/ interv. male			-.033 (.082)	.687		
Age diff. (10) * resp. female/ interv. female			-.113 (.081)	.162		
Age difference (20 years)					.152 (.111)	.170
Age diff. (20) * resp. male/ interv. female					.170 (.153)	.267
Age diff. (20) * resp. female/ interv. male					-.058 (.172)	.734
Age diff. (20) * resp. female/ interv. female					-.461 (.170)	.007
No formal education	Ref. cat		Ref. cat		Ref. cat	
Primary education	.081 (.027)	.003	.079 (.027)	.003	.081 (.027)	.003
Secondary education	.287 (.027)	.000	.284 (.027)	.000	.286 (.029)	.000
Post-secondary education	.459 (.032)	.000	.457 (.032)	.000	.457 (.032)	.000
Christian	Ref. cat		Ref. cat		Ref. cat	
Muslim	-.222 (.028)	.000	-.223 (.027)	.000	-.224 (.028)	.000
Other religion	-.142 (.030)	.000	-.142 (.030)	.000	-.141 (.030)	.000
Urban	.073 (.018)	.000	.073 (.018)	.000	.073 (.018)	.000
Country fixed effects	Yes		Yes		Yes	
Cut-off point 1	.387 (.069)		.390 (.069)		.395 (.068)	
Cut-off point 2	1.070 (.069)		1.070 (.069)		1.075 (.069)	
Cut-off point 3	1.138 (.069)		1.141 (.069)		1.146 (.069)	
Cut-off point 4	2.215 (.069)		2.218 (.069)		2.223 (.069)	
Log likelihood	-66231.749		-66230.193		-66223.722	
LR chi2	5255.97		5259.08		5272.03	
Prob > chi2	.000		.000		.000	
Pseudo Rsquared	.04		.04		.04	
N	51624		51624		51624	

Figures refer to Afrobarometer Round 6.

Table Sm11. Multinomial logistic regression model of the gender of interviewer effects on assessments of whether men make better political leaders than women (multinomial log-odds regression coefficients, standard errors in parentheses)

	'Agree strongly with statement one'		'Agree with statement one'		Neutral mid-category		'Agree with statement two'	
		p		p		p		p
Respondent male/ interviewer male	Ref. cat.		Ref. cat.		Ref. cat.		Ref. cat.	
Respondent male/ interviewer female	-.966 (.036)	.000	-.706 (.041)	.000	-.493 (.106)	.000	-.392 (.033)	.000
Respondent female/ interviewer male	-.527 (.034)	.000	-.586 (.042)	.000	-.281 (.104)	.007	-.333 (.034)	.000
Respondent female/ interviewer female	-1.400 (.038)	.000	-1.209 (.044)	.000	-.915 (.112)	.000	-.645 (.033)	.000
No one present	Ref. cat.		Ref. cat.		Ref. cat.		Ref. cat.	
Spouse present	-.016 (.050)	.753	-.120 (.059)	.056	.079 (.139)	.569	-.025 (.045)	.590
Others present	.034 (.031)	.272	-.099 (.037)	.007	-.077 (.092)	.402	-.093 (.028)	.001
Age difference (15 years)	-.097 (.066)	.142	-.061 (.073)	.402	-.198 (.185)	.286	-.113 (.058)	.052
No formal education	Ref. cat.		Ref. cat.		Ref. cat.		Ref. cat.	
Primary education	-.088 (.040)	.000	-.075 (.049)	.125	-.261 (.122)	.032	-.117 (.039)	.002
Secondary education	-.366 (.041)	.000	-.333 (.050)	.000	-.521 (.123)	.000	-.307 (.039)	.000
Post-secondary education	-.635 (.050)	.000	-.550 (.060)	.000	-.665 (.145)	.000	-.366 (.046)	.000
Christian	Ref. cat		Ref. cat		Ref. cat		Ref. cat	
Muslim	.305 (.042)	.000	.302 (.050)	.000	.028 (.134)	.837	.070 (.040)	.082
Other religion	.176 (.049)	.000	.237 (.054)	.001	.322 (.130)	.013	.094 (.040)	.020
Urban	-.060 (.029)	.036	-.180 (.033)	.000	-.060 (.085)	.477	-.072 (.026)	.005
Constant	1.858 (.109)	.000	.969 (.128)	.000	-.544 (.266)	.041	.932 (.116)	.000
Country fixed effects	Yes		Yes		Yes		Yes	
Log likelihood	-63721.459							
LR chi2	10276.55							
Prob > chi2	.000							
Pseudo Rsquared	.07							
N	51624							

Figures refer to Afrobarometer Round 6. The reference category of the dependent variable is category five, 'Agree strongly with statement two'.

Table Sm12. Generalized ordered logistic regression model of the gender of interviewer effects on assessments of whether men make better political leaders than women (generalized log odds regression coefficients, standard errors in parentheses)

	'Agree strongly with statement one'		'Agree with statement one'		Neutral mid-category		'Agree with statement two'	
		p		p		p		p
Respondent male/ interviewer male	Ref. cat.		Ref. cat.		Ref. cat.		Ref. cat.	
Respondent male/ interviewer female	.720 (.030)	.000	.700 (.027)	.000	.692 (.026)	.000	.654 (.026)	.000
Respondent female/ interviewer male	.270 (.029)	.000	.411 (.026)	.000	.391 (.026)	.000	.442 (.026)	.000
Respondent female/ interviewer female	.946 (.033)	.000	1.060 (.028)	.000	1.049 (.028)	.000	1.001 (.027)	.000
No one present	Ref. cat.		Ref. cat.		Ref. cat.		Ref. cat.	
Spouse present	.005 (.042)	.904	.025 (.038)	.406	.016 (.037)	.667	.057 (.036)	.112
Others present	-.031 (.026)	.245	.009 (.023)	.698	.006 (.023)	.789	.059 (.022)	.007
Age difference (15 years)	.017 (.058)	.774	.023 (.049)	.642	.070 (.049)	.151	.114 (.046)	.017
No formal education	Ref. cat		Ref. cat		Ref. cat		Ref. cat	
Primary education	.019 (.035)	.583	.020 (.031)	.238	.037 (.030)	.110	.114 (.030)	.000
Secondary education	.219 (.036)	.000	.228 (.031)	.000	.241 (.031)	.002	.312 (.030)	.000
Post-secondary education	.427 (.044)	.000	.436 (.038)	.000	.450 (.038)	.000	.482 (.036)	.000
Christian	Ref. cat		Ref. cat		Ref. cat		Ref. cat	
Muslim	-.217 (.035)	.000	-.265 (.032)	.000	-.252 (.031)	.000	-.209 (.031)	.000
Other religion	-.088 (.042)	.037	-.164 (.036)	.000	-.162 (.035)	.000	-.158 (.033)	.000
Urban	.010 (.025)	.674	.062 (.022)	.005	.068 (.022)	.002	.093 (.020)	.000
Constant	-.176 (.080)	.028	-.948 (.078)	.000	-1.156 (.087)	.000	-2.427 (.094)	.000
Country fixed effects	Yes		Yes		Yes		Yes	
'Log likelihood	-63699.237							
LR chi2	10321.00							
Prob > chi2	.000							
Pseudo Rsquared	.08							
N	51624							

Figures refer to Afrobarometer Round 6. The reference category of the dependent variable is category five, 'Agree strongly with statement two'. The model is run with the 'gologit2' command in Stata (Williams 2016).

Table Sm13. Multivariate ordinal logistic regression model of the gender of interviewer effects on assessments of whether men make better political leaders than women, responses coded into three categories (ordered log odds regression coefficients, standard errors in parentheses)

	Model Sm13	
		p
Respondent male/ interviewer male	Ref. cat.	
Respondent male/ interviewer female	.694 (.027)	.000
Respondent female/ interviewer male	.409 (.027)	.000
Respondent female/ interviewer female	1.063 (.029)	.000
No one present	Ref. cat.	
Spouse present	.040 (.038)	.300
Others present	-.014 (.024)	.547
Age difference (15 years)	.040 (.050)	.423
No formal education	Ref. cat	
Primary education	.045 (.032)	.152
Secondary education	.239 (.032)	.000
Post-secondary education	.445 (.039)	.000
Christian	Ref. cat	
Muslim	-.272 (.033)	.000
Other religion	-.171 (.037)	.000
Urban	.083 (.022)	.000
Country fixed effects	Yes	
Cut-off point 1	1.036 (.079)	
Cut-off point 2	1.108 (.079)	
Log likelihood	-33009.668	
LR chi2	5099.46	
Prob > chi2	.000	
Pseudo Rsquared	.07	
N	51624	

Figures refer to Afrobarometer Round 6. The dependent variable is altered so that options on both ends are collapsed into single categories (‘agree’ and ‘strongly agree’ becoming one, for each of the statements).

Table Sm14. Multivariate ordinal logistic regression model of the gender of interviewer effects on assessments of whether men make better political leaders than women, split samples with men and women only (ordered log odds regression coefficients, standard errors in parentheses)

	Model Sm14a (men only)		Model Sm14b (women only)	
		p		p
Interviewer male	Ref. cat.		Ref. cat.	
Interviewer female	.415 (.023)	.000	.331 (.024)	.000
No one present	Ref. cat.		Ref. cat.	
Spouse present	.063 (.043)	.142	-.007 (.049)	.882
Others present	.002 (.029)	.941	.022 (.027)	.420
Age difference (15 years)	.135 (.060)	.024	.010 (.057)	.865
No formal education	Ref. cat		Ref. cat	
Primary education	.095 (.040)	.017	.063 (.037)	.083
Secondary education	.278 (.041)	.000	.311 (.038)	.000
Post-secondary education	.431 (.045)	.000	.519 (.048)	.000
Christian	Ref. cat		Ref. cat	
Muslim	-.249 (.039)	.000	-.187 (.040)	.000
Other religion	-.115 (.039)	.003	-.157 (.045)	.001
Urban	.047 (.026)	.069	.099 (.027)	.000
Country fixed effects	Yes		Yes	
Cut-off point 1	.995 (.102)		-.120 (.101)	
Cut-off point 2	1.672 (.103)		.574 (.101)	
Cut-off point 3	1.743 (.103)		.647 (.101)	
Cut-off point 4	2.791 (.104)		1.770 (.102)	
Log likelihood	-34609.279		-31388.251	
LR chi2	2160.03		2274.27	
Prob > chi2	.000		.000	
Pseudo Rsquared	.03		.04	
N	25726		25898	

Figures refer to Afrobarometer Round 6.

Table Sm15. Multivariate ordinal logistic regression model of the gender of interviewer effects on assessments of whether men make better political leaders than women, split samples with men and women only (ordered log odds regression coefficients, standard errors in parentheses)

	Model Sm15a (men only)		Model Sm15b (women only)	
		p		p
Interviewer male	Ref. cat.		Ref. cat.	
Interviewer female	.676 (.059)	.000	.603 (.051)	.000
No one present	Ref. cat.		Ref. cat.	
Spouse present	.061 (.043)	.153	-.002 (.049)	.968
Others present	.003 (.029)	.911	.021 (.027)	.423
Age difference (15 years)	.129 (.060)	.031	.000 (.057)	1.000
No formal education	Ref. cat		Ref. cat	
Primary education	.219 (.052)	.000	.179 (.048)	.000
Secondary education	.384 (.051)	.000	.475 (.049)	.001
Post-secondary education	.659 (.059)	.000	.796 (.063)	.000
Primary education * interviewer female	-.279 (.074)	.000	-.252 (.067)	.000
Secondary education * interviewer female	-.244 (.070)	.001	-.352 (.066)	.000
Post-second. education * interviewer female	-.493 (.080)	.000	-.581 (.084)	.000
Christian	Ref. cat		Ref. cat	
Muslim	-.250 (.039)	.000	-.181 (.040)	.000
Other religion	-.115 (.039)	.004	.151 (.045)	.001
Urban	.048 (.026)	.051	.098 (.027)	.000
Country fixed effects	Yes		Yes	
Cut-off point 1	.694 (.100)		-.322 (.097)	
Cut-off point 2	1.372 (.100)		.373 (.097)	
Cut-off point 3	1.443 (.100)		.447 (.097)	
Cut-off point 4	2.49 (.101)		1.572 (.098)	
Log likelihood	-34590.096		-31361.424	
LR chi2	2198.40		2327.92	
Prob > chi2	.000		.000	
Pseudo Rsquared	.03		.03	
N	25726		25898	

Figures refer to Afrobarometer Round 6.

Table Sm16. Multivariate ordinal logistic regression models of gender of interviewer effects on whether men make better political leaders: Country-wise models, simplified results (Yes, indicates significance at 95%)

Countries	Respondent male/ interviewer male (reference category)	Respondent male/ interviewer female	Respondent female/ interviewer male	Respondent female/ interviewer female
Algeria	Ref. cat.	Yes	No	Yes
Benin	Ref. cat.	Yes	Yes	Yes
Botswana	Ref. cat.	Yes	Yes	Yes
Burkina Faso	Ref. cat.	Yes	Yes	Yes
Burundi	Ref. cat.	Yes	Yes	Yes
Cameroon	Ref. cat.	Yes	Yes	Yes
Cape Verde	Ref. cat.	Yes	Yes	Yes
Cote d'Ivoir	Ref. cat.	Yes	Yes	Yes
Egypt	Ref. cat.	Yes	No	Yes
Eswatini	Ref. cat.	Yes	Yes	Yes
Gabon	Ref. cat.	Yes	No	Yes
Ghana	Ref. cat.	Yes	Yes	Yes
Guinea	Ref. cat.	Yes	Yes	Yes
Kenya	Ref. cat.	Yes	No	Yes
Lesotho	Ref. cat.	Yes	No	Yes
Liberia	Ref. cat.	Yes	No	Yes
Madagascar	Ref. cat.	Yes	No	Yes
Malawi	Ref. cat.	No	Yes	Yes
Mali	Ref. cat.	Yes	Yes	Yes
Mauritius	Ref. cat.	Yes	No	Yes
Morocco	Ref. cat.	Yes	No	Yes
Mozambique	Ref. cat.	Yes	No	No
Namibia	Ref. cat.	Yes	Yes	Yes
Niger	Ref. cat.	Yes	Yes	Yes
Nigeria	Ref. cat.	Yes	No	Yes
San Tome	Ref. cat.	No	Yes	Yes
Senegal	Ref. cat.	Yes	No	Yes
Sierra Leone	Ref. cat.	Yes	Yes	Yes
South Africa	Ref. cat.	Yes	No	Yes
Sudan	Ref. cat.	Yes	No	Yes
Tanzania	Ref. cat.	Yes	Yes	Yes
Togo	Ref. cat.	Yes	Yes	Yes
Tunisia	Ref. cat.	Yes	Yes	Yes
Uganda	Ref. cat.	Yes	Yes	Yes
Zambia	Ref. cat.	Yes	No	Yes
Zimbabwe	Ref. cat.	Yes	Yes	Yes

For each country in the Afrobarometer survey round 6, we run our main model without controls to see if the interviewer-respondent dyads have an effect on our dependent variable. We denote coefficients as significant with a 'Yes' if $p<0.05$ and 'No' otherwise. Full results for each country are visible in the replication files.

Figure Sm1. Multivariate ordinal logistic regression models of gender of interviewer effects on whether men make better political leaders: Country-wise models, confidence intervals

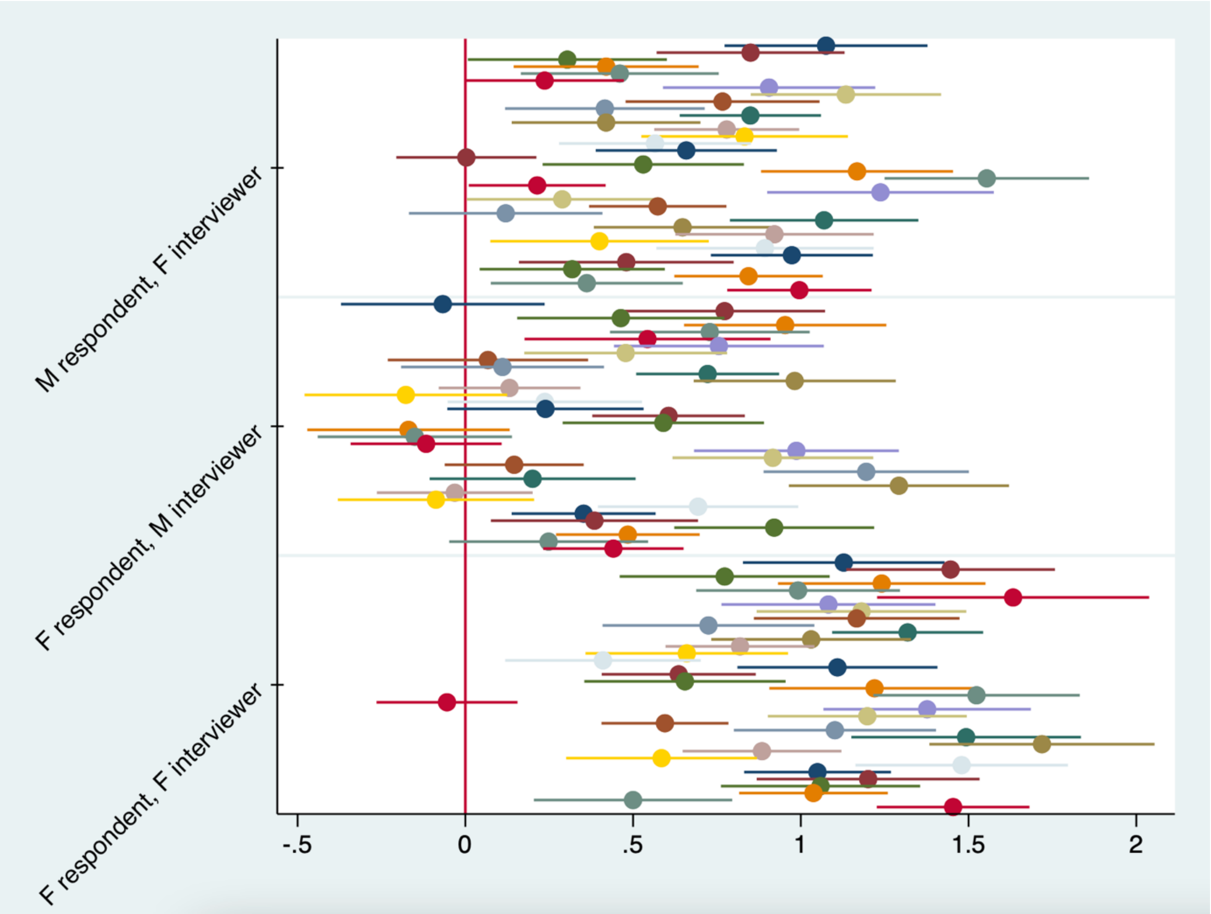

For each country, we run the simple model without controls to see if the interviewer-respondent dyads have an effect on our dependent variable. The figure displays confidence intervals plotted from all 36 countries in the Afrobarometer round 6.

Table Sm17. Multivariate ordinal logistic regression model of the gender of interviewer effects on assessments of whether men make better political leaders than women, alternative fixed-effect specifications (ordered log odds regression coefficients, standard errors in parentheses)

	Model Sm17a		Model Sm17b		Model Sm17c	
				p		p
Respondent male/ interviewer male	Ref. cat.		Ref. cat.		Ref. cat.	
Respondent male/ interviewer female	.678 (.023)	.000	.715 (.024)	.000	.761 (.024)	.000
Respondent female/ interviewer male	.405 (.024)	.000	.420 (.025)	.000	.449 (.026)	.000
Respondent female/ interviewer female	1.017 (.025)	.000	1.058 (.025)	.000	1.129 (.026)	.000
No one present	Ref. cat.		Ref. cat.		Ref. cat.	
Spouse present	.020 (.033)	.552	.017 (.034)	.629	-.012 (.037)	.740
Others present	-.015 (.021)	.478	-.028 (.022)	.201	-.034 (.024)	.144
Age difference (15 years)	.085 (.043)	.048	.112 (.044)	.011	.122 (.048)	.012
No formal education	Ref. cat		Ref. cat		Ref. cat	
Primary education	.062 (.028)	.027	.085 (.029)	.004	.072 (.032)	.000
Secondary education	.271 (.028)	.000	.288 (.030)	.000	.293 (.033)	.000
Post-secondary education	.463 (.034)	.000	.463 (.036)	.000	.462 (.039)	.000
Christian	Ref. cat		Ref. cat		Ref. cat	
Muslim	-.234 (.033)	.000	-.251 (.036)	.000	-.215 (.043)	.000
Other religion	-.113 (.031)	.000	-.121 (.033)	.000	-.128 (.039)	.002
Urban	.042 (.022)	.053	.038 (.029)	.192	-.19.587 (18713.28)	.999
Region fixed effects	Yes					
Local fixed effects			Yes			
Enumeration area fixed effects					Yes	
Cut-off point 1	-1.176 (.189)		.058 (1.126)		-19.773 (18713.28)	
Cut-off point 2	-.480 (.189)		.775 (1.126)		-19.798 (18713.28)	
Cut-off point 3	-.407 (.189)		.851 (1.126)		-19.717 (18713.28)	
Cut-off point 4	.706 (.189)		1.997 (1.126)		-18.463 (18713.28)	
Log likelihood	-65208.084		-62676.154		-60823.375	
LR chi2	7303.30		9502.64		15620.55	
Prob > chi2	.000		.000		.000	
Pseudo Rsquared	.05		.07		.11	
N	51624		50433		51471	

Figures refer to Afrobarometer Round 6. Regional fixed effects account for 431 regions. Local fixed effects account for 2244 units. Model 3 is run on the geocoded Afrobarometer dataset (BenYishay 2018), which contains unique Enumeration Area (EA) information. EA fixed effects account for 7020 units.

Table Sm18. Multivariate ordinal logistic regression model of the gender of interviewer effects on assessments of whether men make better political leaders than women, standard errors clustered on alternate units (ordered log odds regression coefficients, standard errors in parentheses)

	Model Sm18a (Std errors clustered on regions)		Model Sm18b (Std errors clustered on local units)		Model Sm18c (Std errors clustered on enumeration areas)		Model Sm18d (Std errors clustered on interviewers)	
		p		p		p		p
Respondent male/ interviewer male	Ref. cat.		Ref. cat.		Ref. cat.		Ref. cat.	
Respondent male/ interviewer female	.657 (.041)	.000	.667 (.028)	.000	.657 (.022)	.000	.657 (.033)	.000
Respondent female/ interviewer male	.402 (.051)	.000	.401 (.037)	.000	.403 (.024)	.000	.402 (.054)	.000
Respondent female/ interviewer female	.993 (.050)	.000	.998 (.035)	.000	.995 (.024)	.000	.993 (.052)	.000
No one present	Ref. cat.		Ref. cat.		Ref. cat.		Ref. cat.	
Spouse present	.027 (.044)	.526	.027 (.042)	.514	.025 (.033)	.442	.028 (.044)	.534
Others present	.018 (.030)	.548	.011 (.028)	.707	.017 (.021)	.408	.018 (.032)	.571
Age difference (15 years)	.072 (.056)	.201	.071 (.052)	.176	.067 (.043)	.118	.072 (.065)	.266
No formal education	Ref. cat		Ref. cat		Ref. cat		Ref. cat	
Primary education	.080 (.036)	.025	.088 (.031)	.005	.082 (.028)	.004	.080 (.034)	.017
Secondary education	.285 (.041)	.000	.291 (.034)	.000	.286 (.029)	.000	.285 (.037)	.000
Post-secondary education	.458 (.048)	.000	.466 (.041)	.000	.457 (.034)	.000	.458 (.043)	.000
Christian	Ref. cat		Ref. cat		Ref. cat		Ref. cat	
Muslim	-.223 (.045)	.000	-.224 (.039)	.000	-.206 (.031)	.000	-.223 (.049)	.000
Other religion	-.142 (.040)	.000	-.157 (.036)	.000	-.143 (.031)	.000	-.142 (.039)	.000
Urban	.073 (.029)	.012	.079 (.028)	.005	.072 (.021)	.001	.073 (.027)	.007
Country fixed effects	Yes		Yes		Yes		Yes	
Cut-off point 1	.386 (.093)		.399 (.086)		.404 (.076)		.388 (.096)	
Cut-off point 2	1.067 (.092)		1.076 (.084)		1.084 (.076)		1.067 (.094)	
Cut-off point 3	1.139 (.091)		1.148 (.084)		1.154 (.076)		1.139 (.094)	
Cut-off point 4	2.216 (.107)		2.217 (.090)		2.332 (.076)		2.216 (.097)	
Log likelihood	-66230.739		-64875.827		-66010.985		-66230.739	
LR chi2	1691.81		2549.23		4529.21		1904.42	
Prob > chi2	.000		.000		.000		.000	
Pseudo Rsquared	.04		.04		.04		.04	
N	51624		50433		51471		51624	

Model 3 is run on the geocoded Afrobarometer round 6 dataset (BenYishay 2018), which contains unique Enumeration Area (EA) information.

Table Sm19. Multivariate ordinal logistic regression models measuring the gender of interviewer effects on assessments of whether men make better political leaders than women, additional controls of area characteristics (ordered log odds regression coefficients, standard errors in parentheses)

	Model Sm19	
		p
Respondent male/ interviewer male	Ref. cat.	
Respondent male/ interviewer female	.655 (.023)	.000
Respondent female/ interviewer male	.406 (.023)	.000
Respondent female/ interviewer female	.998 (.024)	.000
No one present	Ref. cat.	
Spouse present	.034 (.033)	.298
Others present	.197 (.020)	.329
Age difference (15 years)	.062 (.042)	.137
No formal education	Ref. cat	
Primary education	.076 (.027)	.005
Secondary education	.282 (.027)	.000
Post-secondary education	.449 (.033)	.000
Christian	Ref. cat	
Muslim	-.223 (.028)	.000
Other religion	-.138 (.030)	.000
Urban	.057 (.022)	.009
Sewage system in area	.019 (.025)	.456
Health clinic in area	.033 (.019)	.075
Roadblocks by police/military in area	-.017 (.031)	.587
Country fixed effects	Yes	
Cut-off point 1	.410 (.073)	
Cut-off point 2	1.087 (.073)	
Cut-off point 3	1.158 (.073)	
Cut-off point 4	2.236 (.074)	
Log likelihood	-64940.629	
LR chi2	5164.45	
Prob > chi2	.000	
Pseudo Rsquared	.04	
N	50650	

Figures refer to Afrobarometer Round 6.

Table Sm20. Multivariate ordinal logistic regression model of the gender of interviewer effects on assessments of three gender-related items, Round 5 with a subsample of five Arab countries (Algeria, Egypt, Morocco, Sudan and Tunisia) (ordered log odds regression coefficients, standard errors in parentheses)

	Model Sm20a<br>Attitudes to statement:<br>'Women and men should have equal rights in making a decision to divorce'		Model Sm20b<br>Attitudes to statement:<br>'Women and men should have equal work opportunities'		Model Sm20c<br>Attitudes to statement:<br>'A woman can become the prime minister or president of a Muslim state'	
		p		p		p
Respondent male/ interviewer male	Ref. cat.		Ref. cat.		Ref. cat.	
Respondent male/ interviewer female	.743 (.078)	.000	.820 (.079)	.000	.761 (.077)	.000
Respondent female/ interviewer male	.702 (.073)	.000	.482 (.074)	.000	.407 (.072)	.000
Respondent female/ interviewer female	1.591 (.073)	.000	1.430 (.074)	.000	1.351 (.071)	.000
No one present	Ref. cat.		Ref. cat.		Ref. cat.	
Spouse present	.008 (.083)	.919	.028 (.084)	.741	-.008 (.081)	.925
Others present	.021 (.057)	.708	-.002 (.059)	.970	-.110 (.056)	.052
Age difference (15 years)	.333 (.162)	.040	.615 (.149)	.000	.295 (.146)	.044
No formal education	Ref. cat		Ref. cat		Ref. cat	
Primary education	.183 (.073)	.012	.029 (.074)	.692	-.048 (.072)	.501
Secondary education	.392 (.074)	.000	.090 (.075)	.231	.140 (.072)	.052
Post-secondary education	.520 (.076)	.000	.297 (.077)	.000	.333 (.074)	.000
Christian	Ref. cat		Ref. cat		Ref. cat	
Muslim	-1.353 (.209)	.000	-.684 (.205)	.001	-.864 (.195)	.000
Other religion	-.578 (.438)	.187	-1.497 (.421)	.000	-.538 (.405)	.185
Urban	.275 (.050)	.000	.208 (.051)	.000	.267 (.050)	.000
Country fixed effects	Yes		Yes		Yes	
Cut-off point 1	-1.325 (.236)		-2.999 (.241)		-.706 (.224)	
Cut-off point 2	-.397 (.235)		-1.529 (.235)		.428 (.223)	
Cut-off point 3	.072 (.235)		-1.118 (.234)		.845 (.224)	
Cut-off point 4	1.777 (.237)		.995 (.234)		2.462 (.226)	
Log likelihood	-8187.5106		-7298.2192		-8594.0858	
LR chi2	1255.73		657.42		755.32	
Prob > chi2	.000		.000		.000	
Pseudo Rsquared	.07		.04		.04	
N	5708		5859		5797	

Figures refer to Afrobarometer Round 5. These three items had five response categories; 'Strongly disagree', 'Disagree', 'Neither agree nor disagree', 'Agree' and 'Strongly agree'.

Table Sm21. Multivariate ordinal logistic regression model of gender of interviewer effects on two items: trust in president and view of the economy (ordered log odds regression coefficients, standard errors in parentheses)

	Model Sm21a<br>Attitudes to 'Country's economic condition compared to 12 months ago'		Model Sm21b<br>Attitudes to 'Trust in president'	
		p		p
Respondent male/ interviewer male	Ref. cat.		Ref. cat.	
Respondent male/ interviewer female	.016 (.023)	.489	-.020 (.023)	.373
Respondent female/ interviewer male	-.021 (.023)	.369	-.013 (.023)	.568
Respondent female/ interviewer female	-.013 (.023)	.578	.000 (.023)	.983
No one present	Ref. cat.		Ref. cat.	
Spouse present	-.011 (.031)	.731	.124 (.032)	.000
Others present	.011 (.019)	.585	.018 (.020)	.370
Age difference (15 years)	.103 (.040)	.009	.189 (.041)	.000
No formal education	Ref. cat		Ref. cat	
Primary education	.003 (.027)	.912	-.208 (.028)	.002
Secondary education	.098 (.027)	.000	-.350 (.028)	.000
Post-secondary education	.190 (.032)	.000	-.445 (.032)	.000
Christian	Ref. cat		Ref. cat	
Muslim	.086 (.028)	.002	.142 (.028)	.000
Other religion	.055 (.029)	.057	.008 (.030)	.989
Urban	-.051 (.018)	.004	-.237 (.018)	.000
Country fixed effects	Yes		Yes	
Cut-off point 1	-2.020 (.068)		-1.443 (.069)	
Cut-off point 2	-.403 (.067)		-.306 (.068)	
Cut-off point 3	.810 (.067)		.708 (.068)	
Cut-off point 4	3.178 (.070)			
Log likelihood	-72851.622		-65484.095	
LR chi2	4008.23		7557.70	
Prob > chi2	.000		.000	
Pseudo Rsquared	.03		.05	
N	51031		50873	

Figures refer to Afrobarometer Round 6. These items of the county's economic situation had five response categories; 'Much worse', 'Worse, 'Same', 'Better' and 'Much better'. The item of trust in president had four response categories; 'Not at all', 'Just a little, 'Somewhat', and 'a lot'.

Table Sm22. Exact question wording of items used, Afrobarometer round 6, information from English version codebook

Item	Question wording
	Question: Which of the following statements is closest to your view? Choose Statement 1 or Statement 2. Statement 1: Men make better political leaders than women, and should be elected rather than women. Statement 2: Women should have the same chance of being elected to political office as men.<br>*Values:* 1=Agree very strongly with Statement 1, 2=Agree with Statement 1, 3=Agree with Statement 2, 4=Agree very strongly with Statement 2, 5=Agree with neither, 9=Don't know, 98=Refused to answer, - 1=Missing<br>*Note:* Interviewer probed for strength of opinion asking "Do you agree or agree very strongly?"
Interviewer gender	*Question:* Interviewer's gender<br>*Values:* 1=Male, 2=Female<br>*Note:* Answered by interviewer
Respondent gender	*Question:* Respondent's gender<br>*Values:* 1=Male, 2=Female<br>*Note:* Answered by interviewer
Presence of spouse or others	*Question:* Were there any other people immediately present who might be listening during the interview?<br>*Values:* 1=No one, 2=Spouse only, 3=Children only, 4=A few others, 5=Small crowd, - 1=Missing<br>*Note:* Answered by interviewer
Respondent age	*Question:* How old are you?<br>*Values:* 18-105, 998-999, -1 Value Labels: 98=Refused to answer, 999=Don't know, - 1=Missing
Interviewer age	*Question:* Interviewer's age<br>*Values:* 16-62<br>*Note:* Answered by interviewer
Education	*Question:* What is your highest level of education?<br>*Value:* 0=No formal schooling, 1=Informal schooling only (including Koranic schooling), 2=Some primary schooling, 3=Primary school completed, 4=Intermediate school or Some secondary school / high school, 5=Secondary school / high school completed , 6=Post-secondary qualifications, other than university e.g. a diploma or degree from a polytechnic or college, 7=Some university, 8=University completed, 9=Post-graduate, 99=Don't know, 98=Refused to answer, -1=Missing
Religion	*Question*: What is your religion, if any?<br>*Values*: 0-34, 100, 220, 260,<br>*Note*: variable recoded by the Afrobarometer from numerous response categories to a variable with the categories 'Christians', 'Muslims' and 'other religions'
Urban/ rural	*Question*: Urban or rural primary sampling unit<br>*Values*: 1=urban, 2=rural<br>*Note*: Answered by interviewer

For more information, see the Afrobarometer round 6 codebook, at: https://afrobarometer.org/data/merged-round-6-codebook-36-countries-2016
